# The N-Acetyl Phenylalanine Glucosamine Derivative Attenuates the Inflammatory/Catabolic Environment in a Chondrocyte-Synoviocyte Co-Culture System

**DOI:** 10.1038/s41598-019-49188-9

**Published:** 2019-09-19

**Authors:** Stefania Pagani, Manuela Minguzzi, Laura Sicuro, Francesca Veronesi, Spartaco Santi, Anna Scotto D’Abusco, Milena Fini, Rosa Maria Borzì

**Affiliations:** 1 Laboratory of Preclinical and Surgical Studies, IRCCS Istituto Ortopedico Rizzoli, via di Barbiano 1/10, 40136 Bologna, Italy; 2 Department of Medical and Surgical Sciences “Alma Mater Studiorum” University of Bologna, via Massarenti 9, 40138 Bologna, Italy; 3 Laboratory of Immunorheumatology and Tissue Regeneration, IRCCS Istituto Ortopedico Rizzoli, via di Barbiano 1/10, 40136 Bologna, Italy; 4 Institute of Molecular Genetics, National Research Council (CNR), 40136 Bologna, Italy; 5 IRCCS Istituto Ortopedico Rizzoli, via di Barbiano 1/10, 40136 Bologna, Italy; 6 Department of Biochemical Sciences, Sapienza University of Roma, P.le Aldo Moro 5, Roma, 00185 Italy

**Keywords:** Mechanisms of disease, Osteoarthritis

## Abstract

Osteoarthritis (OA), the most prevalent degenerative joint disease, still lacks a true disease-modifying therapy. The involvement of the NF-κB pathway and its upstream activating kinases in OA pathogenesis has been recognized for many years. The ability of the N-acetyl phenylalanine glucosamine derivative (NAPA) to increase anabolism and reduce catabolism via inhibition of IKKα kinase has been previously observed *in vitro* and *in vivo*. The present study aims to confirm the chondroprotective effects of NAPA in an *in vitro* model of joint OA established with primary cells, respecting both the crosstalk between chondrocytes and synoviocytes and their phenotypes. This model satisfactorily reproduces some features of the previously investigated DMM model, such as the prominent induction of ADAMTS-5 upon inflammatory stimulation. Both gene and protein expression analysis indicated the ability of NAPA to counteract key cartilage catabolic enzymes (ADAMTS-5) and effectors (MCP-1). Molecular analysis showed the ability of NAPA to reduce IKKα nuclear translocation and H3Ser10 phosphorylation, thus inhibiting IKKα transactivation of NF-κB signalling, a pivotal step in the NF-κB-dependent gene expression of some of its targets. In conclusion, our data confirm that NAPA could truly act as a disease-modifying drug in OA.

## Introduction

Osteoarthritis (OA), considered for many decades a chronic degenerative disease and more recently, an inflammatory disease, is the most diffuse rheumatic disease, affecting up to 15% of the adult population worldwide^1^. OA is characterized by a progressive degradation of the extracellular matrix (ECM) of articular cartilage until its complete erosion and uncovering of the subchondral bone (SB)^2^. The vicious cycle that is established starts with a weak matrix alteration, the subsequent response by chondrocytes and synoviocytes in terms of pro-inflammatory cytokine release, the activation of some transcription factors, and finally the production of catabolic mediators such as metalloproteases (MMPs) and aggrecanases. The increasing inflammatory state leads to an imbalance between the synthesis and degradation of extracellular matrix components. This inflammation leads to poor quality of life, pain and decreased joint functionality for the patients^3–5^.

Even if some mechanisms underlying cartilage destruction are known, many are still not clearly identified. Among the key players of intracellular pathways, a well-known actor is nuclear factor kappa-light-chain-enhancer of activated B cells (NF-κB). NF-κB is a complex of five different subunits that acts as a transcription factor by the assembly of alternative homodimers and heterodimers, thus inducing gene transcription of many inflammatory cytokines. The removal of IκB inhibitors from the cytoplasmic NF-κB complex by the activated kinases IKKα or IKKβ allows the translocation of NF-κB dimers into the nucleus and the consequent transcription of inflammatory mediators and catabolic enzymes, such as interleukin (IL)-6, IL-8, and MMPs^6,7^. A prolonged inflammatory state provokes the loss of the growth-arrested state of articular chondrocytes, deregulated gene expression, and consequent cartilage degradation^8^.

Despite the widespread occurrence of OA, to date, a therapy able to heal this chronic pathology is lacking. The current management of patients includes physical exercise, rehabilitation, and known pharmacological agents: analgesics, opioids, non-steroidal anti-inflammatory drugs (NSAIDs), and corticoids. However, these approaches can only treat symptoms, rather than the underlying process, at best improving functionality and pain^7,9^. Therefore, the need to develop therapies aimed at preventing, delaying or defusing joint inflammation and degenerative progression remains great.

As a possible alternative, several chondroprotective agents have been recently studied: glucosamine (GlcN), chondroitin sulphate, diacerein and curcumin^10–12^, in addition to hyaluronic acid (HA) for viscosupplementation intervention^9^.

Several *in vitro* studies have already underlined the positive effects of GlcN, an essential compound of most cartilage proteoglycans; it inhibits NF-κB and activator protein 1 (AP-1) activation^13,14^, IL-6, iNOS, COX-2^12^, and PGE2 release^15,16^, MMPs and collagenase activity^17^, while stimulating aggrecan synthesis^18^. However, in addition to a large body of encouraging evidence that supported decades of clinical use of GlcN as a treatment for OA, negative or contradictory effects have also been reported, both *in vitro*^17,19^ and *in vivo* ^20–22^.

In this regard, the synthesis and selection of 2-(N-acetyl)-L-phenylalanylamido-2-deoxy-β-D-glucose (NAPA), among the GlcN derivatives, was related to its higher bioavailability compared with GlcN at the intracellular level, due to the hydrophobic character imparted by the aromatic ring of phenylalanine. NAPA seems to exert better anti-inflammatory and chondroprotective activity compared to GlcN, according to recent studies highlighting their differential roles in the intracellular inflammatory pathway^5,13^.

The results obtained with human and rabbit primary and immortalized chondrocytes showed interesting effects on inflammatory cytokines under NF-κB control, such as IL-6 and IL-8^5^. Both GlcN and NAPA effectively counteracted the negative effects of IL-1β and TNFα administration, reducing matrix metalloproteinase production and reverting the gene expression of IL-6 and IL-8. Conversely, the inhibition of IKKα kinase activity was exerted only by NAPA, whereas the inhibition of IKKα nuclear re-localization due to both chondroprotective compounds^5^.

We recently reported the effectiveness of intra-articular delivery of NAPA in reducing OA progression in a murine surgical OA model obtained by destabilization of the medial meniscus (DMM)^7^. The *in vivo* studies demonstrated the efficacy of NAPA for OA treatment as evaluated by histology, histomorphometry and immunohistochemical assessment of some selected markers (MMP-13, ADAMTS5, MMP-10 and IKKα).

Notably, in the *in vivo* model, NAPA administration was performed at 4 weeks post-DMM, a time that corresponds to a mild-to-moderate OA (4 weeks), i.e., a disease stage that in human OA could be more amenable to disease-modifying treatments compared to the moderate-to-severe stage. This setting can be mimicked *in vitro* by sustained stimulation with IL-1β that, in our experimental design, preceded the addition of NAPA. Interestingly, in OA onset and development, a crucial role is played by the synovial-to-cartilage crosstalk^23^ that we aimed to reproduce in the present paper using a co-culture system. Therefore, compared to other *in vitro* studies, the present study proposed a different culture system and stimulation timing to develop a more realistic model, which is the closest possible to our previous *in vivo* study. NAPA administration was performed some days after inflammatory induction to better reproduce the real timing of a pharmacological treatment to a joint at the stage known as “early OA”.

Chondrocytes and synoviocytes, derived from either healthy or OA donors, were used and kept in co-culture. To explore the effects of the inflammatory stimulus and NAPA treatment in a “site specific manner”, we evaluated both chondrocytes and synoviocytes for changes in gene expression, while analysis of protein content was possible only for chondrocytes that were evaluated for changes in signalling and effector molecules. Moreover, our analysis included the cumulative contribution of chondrocytes and synoviocytes to the supernatant, thus mimicking a synovial fluid-like environment. Pivotal inflammatory and matrix degrading molecules were investigated both at the level of gene transcription (in both chondrocytes and synoviocytes) and protein release. To gain further insight into NAPA activity, we explored the underlying molecular mechanisms from the perspective of translating the findings to the treatment of human OA. Therefore, the effects on chondrocytes of NAPA delivery to co-cultures were evaluated by immunofluorescence to assess the level of IKKα nuclear expression and by both western blot and immunofluorescence to determine the extent of phosphorylated serine 10 of histone H3.

## Materials and Methods

### Cell cultures

Experiments were performed with commercially available chondrocytes and synoviocytes of either healthy or OA origin, in accordance with relevant guidelines and regulations (Rizzoli Orthopaedic Institute has kept the ISO 9001 Quality certification since 2008, with special reference to the Research area). Co-cultures of primary chondrocytes and synoviocytes were set up in 24-well plates to test the anti-inflammatory effects of NAPA.

First, NHC-a (normal human chondrocytes –adult) or HC-OA (human chondrocytes-osteoarthritis) purchased from Cell Application (San Diego, CA, USA) were seeded at a density of 5 × 10^4^/cm^2^ on the well bottom, maintained in complete differentiating medium (CDBM, LONZA Walkersville, MD, USA) containing 5% foetal bovine serum (FBS), supplemented with 100 U/ml penicillin and 100 μg/ml streptomycin (Sigma-Aldrich, Arklow, Ireland), and cultured for 1 week to reach a hyperconfluent status^24^.

After reaching this status, HFLS (human fibroblast-like synoviocytes) or HFLS-OA (human fibroblast-like synoviocytes –osteoarthritis) (Cell Application) were seeded at a density of 4 × 10^4^/cm^2^ onto the bottom of a polyester cell culture insert (COSTAR, Corning Incorporated, Kennebunk, ME, USA; surface area 0.33 cm^2^) in a mixture of Dulbecco’s Modified Eagle’s Medium (DMEM) and F12 supplemented with 10% FBS.

The following day, the co-cultures were assembled, and the inflammatory stimulation (10 ng/ml recombinant interleukin (IL)-1β (PeproTech EC Ltd., London, UK)) was started in two-thirds of the wells and maintained for one week. Then, after changing the medium, the IL-β-treated wells were again maintained with 10 ng/ml IL-1β, but half of these IL-1β-treated wells were also treated with NAPA; its effects were investigated at 0, 8, 24 and 48 hours. Therefore, the cells left untreated for one week and after the medium change represented the control (CTR), and the cells treated with IL-1β for 1 week and after the medium change represented the inflammatory condition (IL-1β), while the cells treated for 1 week with IL-1β and subsequently treated with a single dose of 5 mM NAPA, in addition to IL-1β, represented the treated condition (NAPA). Each condition was carried out in triplicate wells.

Three independent experiments were carried out with normal cells, and two independent experiments were carried out with OA cells.

All evaluations were performed at the time of NAPA administration (T zero or basal) and after 8, 24 and 48 hours.

### Cell viability

To evaluate cell viability at each time-point, the cultures of chondrocytes and synoviocytes were disassembled, and Alamar blue dye (Serotec, Oxford, UK) was added to the media of CTR and experimental conditions (1:10 v/v) and incubated for 4 hours at 37 °C.

The reagent is a dye that incorporates an oxidation-reduction (REDOX) indicator that changes colour in response to chemical reduction of the growth medium that is proportional to cell growth. The fluorescence was read at 530ex-590em nm wavelengths by a Micro Plate reader (VICTOR X2030, Perkin Elmer, Milan, Italy) and expressed as relative fluorescence units (RFU). For each experiment, the data were normalized for the values of the non-stimulated control at T0.

### Gene expression

Total RNA was extracted separately from chondrocytes and synoviocytes with the PureLink RNA mini kit (Ambion), quantified by a NANODROP spectrophotometer (NANODROP 2720, Thermal Cycler, Applied Biosystem) and reverse transcribed with the Superscript VILO cDNA Synthesis kit (Life Technologies) according to the manufacturer’s instructions. Each sample was diluted to a final concentration of 5 ng/μl, taking into account the starting amount of RNA, to exploit the same range of amplification efficiency. Gene expression was evaluated by semi-quantitative PCR analysis using the SYBR Green PCR kit (Qiagen) in a Light Cycler 2.0 instrument (Roche Diagnostics). Five nanograms of each sample was tested in duplicate. The protocol included a denaturation cycle at 95 °C for 15 min, 25–40 cycles of amplification (95 °C for 15 s, appropriate annealing temperature for each target, as detailed in Table 1, for 20 s, and 72 °C for 20 s) and a melting curve analysis to check for amplicon specificity.

**Table 1:** Details of primers used for gene expression analysis.

GENE	Forward primer	Reverse primer	Amplicon Length	Annealing Temperature	Cell type
GAPDH	5′-TGGTATCGTGGAAGGACTCA-3′	5′-GCAGGGATGATGTTCTGGA -3′	123 bp	56 °C	C, S
MMP1	5′-GACAGAGATGAAGTCCGGTTT-3′	5′-GCCAAAGGAGCTGTAGATGTC-3′	102 bp	60 °C	C, S
MMP3	5′-CACAGACCTGACTCGGTTCC-3′	5′-AAGCAGGATCACAGTTGGCT-3′	152 bp	60 °C	C, S
MMP8	5′-GCTTCCATTTCTGCTCTTACTC-3′	5′-GCCATTCTTCCTTGTAGACTGA-3′	215 bp	60 °C	C
MMP10	5′-GCCAGTCCATGGAGCAAGGCT-3′	5′-TCGCCTAGCAATGTAACCAGCTGT-3′	195 bp	58 °C	C, S
MMP13	5′-AGCCACTTTATGCTTCCTGA-3′	5′-TGGCATCAAGGGATAAGGAAG-3′	130 bp	60 °C	C, S
ADAMTS 4	5′-CTGCCTACAACCACCG-3′	5′-GCAACCAGAACCGTCC-3′	293 bp	58 °C	C, S
ADAMTS 5	5′-GCACTTCAGCCACCATCAC-3′	5′-AGGCGAGCACAGACATCC-3′	187 bp	58 °C	C, S
TIMP 3	5′-CCTTGGCTCGGGCTCATC-3′	5′-GGATCACGATGTCGGAGTTG-3′	121 bp	60 °C	C
IL 1β	QuantiTect Primer Assay (Qiagen) Hs_IL1B_1_SG		117 bp	55 °C'	C
IL 6	5′-GCAGATGAGTACAAAAGTCCTGA-3′	5′-TTCTGTGCCTGCAGCTTC-3′	120 bp	60 °C	C, S
IL 8	5′-ATGACTTCCAAGCTGGCCGTG-3′	5′-TTATGAATTCTCAGCCCTCTTCAAAAACTTCTC-3′	300 bp	60 °C	C, S
IL 10	5′-GCGCTGTCATCGATTTCTTC-3′	5′-TCACTCATGGCTTTGTAGATGC-3′	108 bp	60 °C	S
TNFα	QuantiTect Primer Assay (Qiagen) Hs_TNF_1_SG		104 bp	55 °C	C, S
GROα	5′-ATTCACCCCAAGAACATCC-3′	5′-GATTTGTCACTGTTCAGCATC-3′	164 bp	56 °C	C
MCP1	5′-GAAGCTCGCACTCTCGCCT-3′	5′-GAGTGTTCAAGTCTTCGGA-3′	330 bp	56 °C	C,S
RANTES	5′-AGGTACCATGAAGGTCTCC-3′	5′-GACTCTCCATCCTAGCTCA-3′	294 bp	56 °C	C,S
IKKα	QuantiTect Primer Assay (Qiagen) Hs_CHUK_1_SG		109 bp	55 °C	C
NFKB1	5′-CAGGAGACGTGAAGATGCTG-3'	5′-AGTTGAGAATGAAGGTGGATGA-3'	109 bp	60 °C	C,S
RELA	5′-CGAGCTTGTAGGAAAGGACTG-3'	5′-TGACTGATAGCCTGCTCCAG-3'	132 bp	60 °C	C,S
NFKBIA	5′-TCCTGAAGGCTACCAACTACA-3'	5′-CATTGACATCAGCACCCAAG-3'	108 bp	60 °C	C,S
iNOS 2	QuantiTect Primer Assay (Qiagen) Hs_NOS2_1_SG		92 bp	55 °C	C
SOX 9	5′-GAGCAGACGCACATCTC-3′	5′-CCTGGGATTGCCCCGA-3′	118 bp	60 °C	C
ACAN	5′-TCGAGGACAGCGAGGCC-3′	5′-TCGAGGGTGTAGCGTGTAGAGA-3′	85 bp	60 °C	C
COL2A1	GTTTTCCAGCTTCACCATCATC	CCTCAAGGATTTCAAGGCAAT	121 bp	60 °C	C

The letters C: chondrocytes and S: synoviocytes, specify the cell type in which the gene expression was analyzed.

In an effort to correlate NAPA activity with gene expression, a broad spectrum of genes has been analysed in both cellular types. To better appreciate the trend of RNA accumulation and the basal level of expression, we chose to express transcription by the 2^−ΔCt^ method^25^ and presented the results in terms of the number of molecules of the gene of interest per 100000 molecules of the housekeeping gene GAPDH. Each evaluation was performed on three replicate cultures. The extracted RNA was pooled and processed. Each experimental condition was then tested in duplicate.

### Protein release

The supernatants harvested at all time points from the triplicate wells containing the co-cultures of chondrocytes and synoviocytes were centrifuged to eliminate particulates and maintained at −80 °C until the evaluation. Immunoenzymatic assays (ELISA) were employed to quantify the amount of Tumour Necrosis Factor α (TNFα) and Interleukin 6 (IL-6), (CLOUD USCN Life Science), according to the manufacturer’s instructions. Moreover, the release in the supernatants of a panel of four chemokines (CCL-2/MCP-1, CCL-5/RANTES, CCL-3/MIP-1α and CCL-4/MIP-1β) and a wide panel of metalloproteases (MMP-1, MMP-2, MMP-3, MMP-7, MMP-8, MMP-9, MMP-10, MMP-12, MMP-13) was quantified by using a Bio-Plex Pro assay (BIO-RAD Laboratories) that exploits a bead-based sandwich immunoassay technology with high sensitivity and dynamic range.

### Western blot

To achieve effective extraction of both nuclear and cytoplasmic proteins, including those bound to DNA, radioimmunoprecipitation (RIPA) buffer with the addition of benzonase and protease inhibitor cocktail (PIC; Sigma-Aldrich) was used to extract proteins from the pellets of the cells recovered from each well. The composition of the buffer was as follows: 50 mM Tris-HCl pH 7.4, 150 mM NaCl, 1% Nonidet P-40, 0.1% SDS, 0.5% Na deoxycholate, 1 mM NaF, 1 mM Na_3_VO_4_, 1 mM PMSF, 1:200 PIC, and 100 U/ml benzonase. Briefly, total cellular lysates were obtained by solubilizing the cells with RIPA buffer in addition to vigorous homogenization with disposable pestles (Sigma) and vortexing.

A volume of lysate corresponding to 160000 cells was loaded in the wells of Nu-Page precast 4–10% polyacrylamide gels (Invitrogen), which were subsequently transferred onto polyvinylidene fluoride membranes by a dry electroblotting method using an I-Blot (Invitrogen). Then, the blots were subjected to immunodetection using a SNAP-ID 2.0 device (Merck Millipore). Signals were detected with appropriate secondary antibodies and visualized with ECL Select (Amersham) using the CCD camera acquisition system of a ChemiDoc Imaging Systems apparatus (BioRad). Western blot images were then analysed by means of a Carestream Molecular Imaging Software 5.0 (Carestream Health, Inc.) to accurately and automatically assess the molecular weight of the bands, using a proper molecular weight marker (Novex Sharp Pre-stained Protein Standard, Invitrogen). Semi-quantitative analysis of band intensity was performed considering “optical density” values and using QuantityOne software (BioRad).

The following primary antibodies were used: anti-phospho-histone H3, (Ser10), (mouse monoclonal, Upstate–Millipore); anti-ADAMTS5, (rabbit polyclonal, Millipore); and anti-NFKB1 (p105/p50) (rabbit polyclonal, Abcam). Anti-GAPDH (mouse monoclonal, Merck Millipore) served as a loading control. Appropriate anti-species antibodies and HRP-conjugated antibodies were from Jackson laboratories.

### Immunofluorescence

Experiments were carried out to better appreciate the subcellular IKKα distribution in chondrocytes in all the previously described conditions.

At first, this analysis was carried out with cells cultured in parallel with those used for gene expression and protein analysis, i.e., in 24-well plates. Images of these experiments were observed by means of an inverted Nikon Eclipse TiU equipped with 530ex/580em filters to evaluate IKKα stained with a red emitting fluorochrome, while nuclear counterstaining with DAPI was evaluated with 360ex/460em filters. Additional experiments were performed to observe the IKKα subcellular distribution in conjunction with the extent of phosphorylation of Ser10 in Histone H3 by double immunofluorescence labelling. For this purpose, chondrocytes were seeded onto round coverslips placed in the wells of a 24-well plate to subsequently permit glass removal, processing and image acquisition by means of both a NIKON Eclipse 90i fluorescence microscope and a NIKON confocal microscope system A1, essentially as previously described^26^. The hyperconfluent cultures of chondrocytes under all conditions were washed with PBS (phosphate-buffered saline) and fixed with 4% paraformaldehyde for 30 min at RT. The samples were pre-treated for antigen unmasking with 0.02 U/ml Chondroitinase ABC (SIGMA) in 50 mM Tris/HCl pH 8.0 for 20 min at 37 °C and permeabilized with 0.2% Triton in TBS (TRIS-buffered saline) solution for 5 min at RT. After extensive washes with TBS, the non-specific bindings were blocked with 5% BSA (bovine serum albumin), 5% normal donkey serum and 0.1% Triton in TBS for 30 min at RT and washed again. IKKα staining was performed with 5 µg/ml rabbit anti-IKKα antibody (BOSTER Biological Technology, Freemont, CA, USA), while the extent of phosphorylation of serine 10 of histone H3 was evaluated with 5 µg/ml anti-phospho-histone H3, (Ser10), (mouse monoclonal, Upstate–Millipore) overnight at 4 °C. After rinsing in TBS, the signal was visualized with 15 µg/ml Alexa Fluor® 555 donkey anti-rabbit and 15 µg/ml Alexa Fluor® 488 donkey anti-mouse IgG secondary antibody conjugates (Novex), incubated for 30 min at RT together with 1 µg/ml Hoechst 33342 (Sigma Aldrich) for nuclear counterstaining. Finally, the samples were mounted with the addition of anti-fading reagent (1% 1,4 Diazobicyclo (2.2.2) Octane (DABCO, SIGMA) in 90% glycerol in 0.1 M pH 8.0 Tris-HCl), sealed with nail polish and stored refrigerated and in the dark for subsequent analysis.

The acquisition of Alexa 488-labelled anti-hIKKα antibody, Alexa 555-labelled anti-phospho-histone H3 (Ser10) and Hoechst 33342-labelled DNA signals was also performed using an A1-R confocal laser scanning microscope (Nikon), equipped with a Nikon 60x, 1.4 NA objective, and with 405, 488 and 561 nm laser lines to excite Hoechst 33342 (blue), Alexa 488 (green) and Alexa 555 (red) fluorescence signals, respectively.

Emission signals were detected by a photomultiplier tube (DU4) preceded by emission filters BP 525/50 nm and BP 595/50 nm for Alexa Fluor 488 and Alexa Fluor 555, respectively. Laser scanning, image acquisition and processing were performed with Nikon Imaging Software NIS Elements AR-4 (Nikon Inc., USA). Optical sections were spaced *0.3 µm along the z axis and were digitized with a scanning mode format of 1024 × 1024 pixels and 4096 grey levels. Both optical sections and 3D projection images were obtained.

### Statistics

The graphs represent the cumulative analysis of at least four different experiments or otherwise stated in the Figure Legend. All data are presented as the mean ± standard deviation (SD) and analysed and graphed using GraphPad Prism version 5.00 for Windows (GraphPad Software).

An initial screening was carried out at time 0 to compare each IL-1β condition versus the paired CTR condition. The means of groups were compared with two-tailed Student’s t test for paired samples, and the differences were considered significant when P < 0.05, with *P < 0.05; **P < 0.01; and ***P < 0.001. Then, at each time point of NAPA treatment (8, 24 and 48 hours), since comparisons were undertaken among multiple groups (CTR, IL-1β-treated and IL-1β + NAPA-treated samples), ANOVA was used, followed by Tukey’s post hoc test. Again, the differences were considered significant when P < 0.05 when comparing multiple groups, but different symbols were used for different comparisons: #P < 0.05; ##P < 0.01; and ###P < 0.001 were used to score the degree of significance of the differences of either IL-1β treatment or IL-1β + NAPA treatment compared to the control condition and *P < 0.05; **P < 0.01; and ***P < 0.001 were used for IL-1β treatment compared to IL-1β + NAPA treatment.

## Results

### IL-1β or NAPA treatment does not affect cell viability

**Figure 1:**
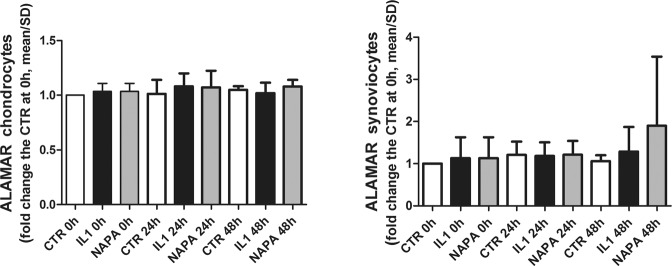
IL-1β or NAPA treatments do not affect cell viability. At each time point, before extraction for gene expression analysis, the co-cultures of chondrocytes and synoviocytes were disassembled, and Alamar blue dye was added to the media of CTR and treated samples (experimental conditions) (1:10 v/v) and incubated for 4 hours at 37 °C. Then, the fluorescence was read, and the values for each experiment (expressed as relative fluorescence units) were normalized for the values of the unstimulated control at T0. The left graph shows chondrocyte viability, while the right graph shows synoviocyte viability (mean ± standard deviation, n = 4).

The viability assessment performed on both chondrocyte and synoviocyte cultures used for the co-culture setting did not reveal significant differences among the groups for both healthy and osteoarthritic cells or across the different time points (Fig. 1). This observation allowed us to exclude toxic effects of NAPA on the cultures, in keeping with previous findings^5^. Moreover, the additional evidence that the different treatments produced similar effects on both healthy and OA chondrocytes allowed us to present the results cumulatively, as described thereafter.

### IL-1β delivery to co-cultures of chondrocytes and synoviocytes reproduces an OA joint environment, and NAPA reduces the expression of pivotal OA effectors

- Gene expression

#### IL-1β strongly upregulates gene expression of catabolic and inflammatory genes in chondrocytes and synoviocytes

Based on the known anti-inflammatory activity of GlcN, which is able to modulate the NF-κB signalling pathway and its target genes, the effect of its NAPA derivative was at first assessed on the expression of a wide range of NF-κB-dependent genes with a pivotal role in OA pathophysiology, including catabolic enzymes and inflammatory molecules (cytokines and chemokines). A selection of genes was investigated in both chondrocytes and synoviocytes, as detailed in Table 1. Other genes were only assessed in chondrocytes since their higher number allowed for a higher cDNA yield.

IL-1β markedly increased the expression of most of the analysed genes at T0 (Supplementary Figs 1, 2 and 3), thus confirming the suitability of the *in vitro* model. Some genes were found to be elevated by IL-1β treatment in both chondrocytes and synoviocytes. IL-1β significantly increased chondrocyte expression of many catabolic enzymes (Supplementary Fig. 1: MMP-3, MMP-10, MMP-1, and ADAMTS5), cytokines and chemokines (Supplementary Fig. 2: IL-1β, TNFα, IL-6, MCP-1, IL-8 and GROα) and NFKB1 (Supplementary Fig. 3), a protein that is both an NF-κB member and a known NF-κB target gene^27^, thus further confirming that culture conditions elicited NF-κB activation. Moreover, despite failing to reach statistical significance, IL-1β stimulation led to reduced expression of Sox-9 and collagen 2A1, as previously reported^28^. The expression of TNFα and IL-6 was also significantly increased in synoviocytes.

#### NAPA reduces the gene expression of key OA effectors in chondrocytes and synoviocytes

**Figure 2:**
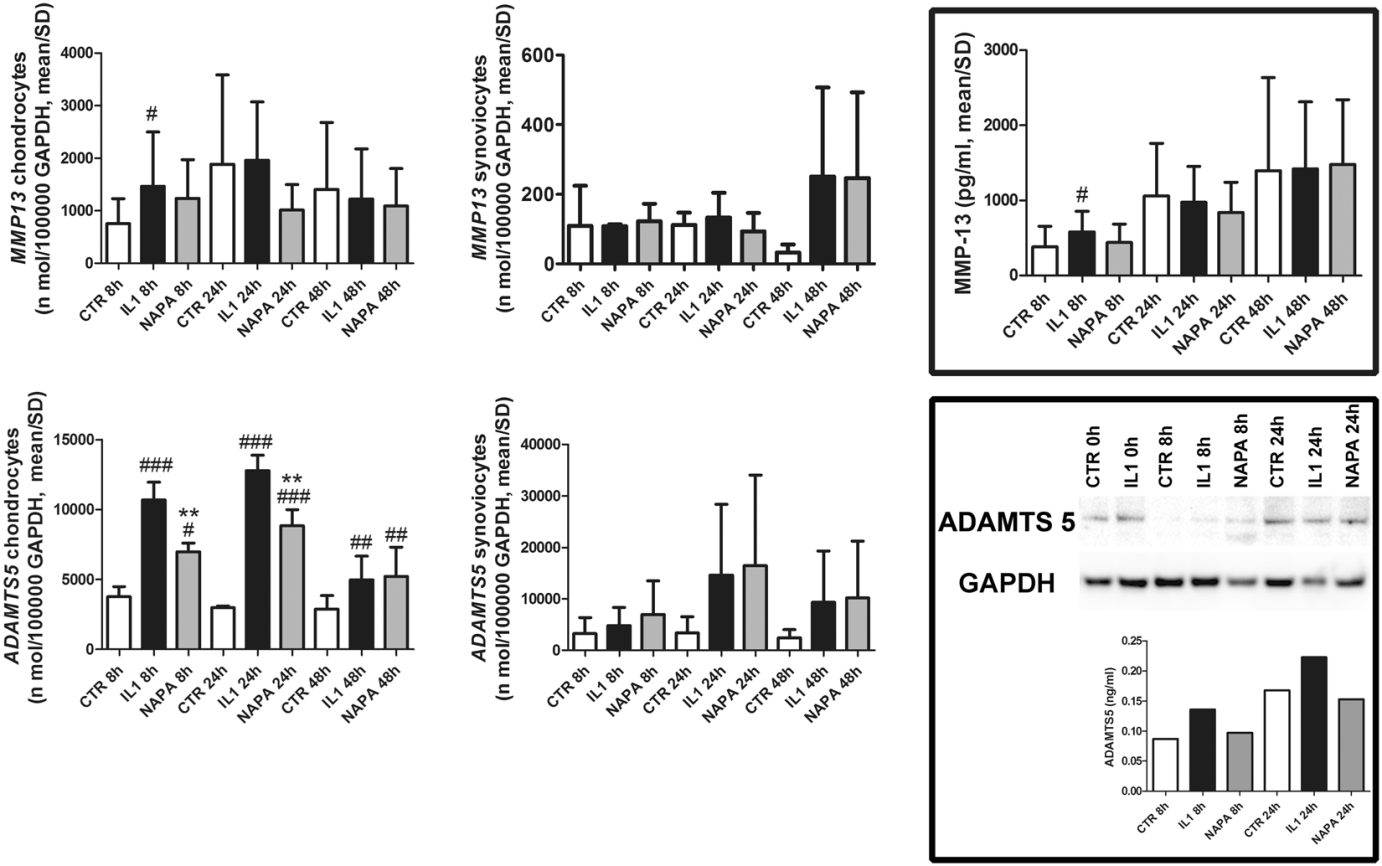
IL-1β strongly upregulates gene expression of pivotal catabolic genes in chondrocytes, and NAPA counteracts this effect. Left graphs: MMP-13 and ADAMTS5 gene expression in chondrocytes; middle graphs: MMP-13 and ADAMTS5 gene expression in synoviocytes; right framed graphs: protein assessment of MMP-13 in the supernatant (above) and of ADAMTS5 (below) in both chondrocyte extract and co-culture supernatant [mean ± standard deviation; chondrocytes: n = 8 (4 experiments in duplicate); synoviocytes: n = 4 (2 experiments in duplicate)]. At each time point of NAPA treatment (8, 24 and 48 hours), the means of groups (CTR, IL-1β treated and IL-1β + NAPA treated samples) were compared by ANOVA, followed by Tukey’s post hoc test. Different symbols were used for different comparisons: ^#^P < 0.05; ^##^P < 0.01 and ^###^P < 0.001 were used to score the degree of significance of the differences of either IL-1β treatment or IL-1β + NAPA treatment compared to the control condition and *P < 0.05; **P < 0.01; and ***P < 0.001 for IL-1β treatment compared to the IL-1β + NAPA treatment.

**Figure 3:**
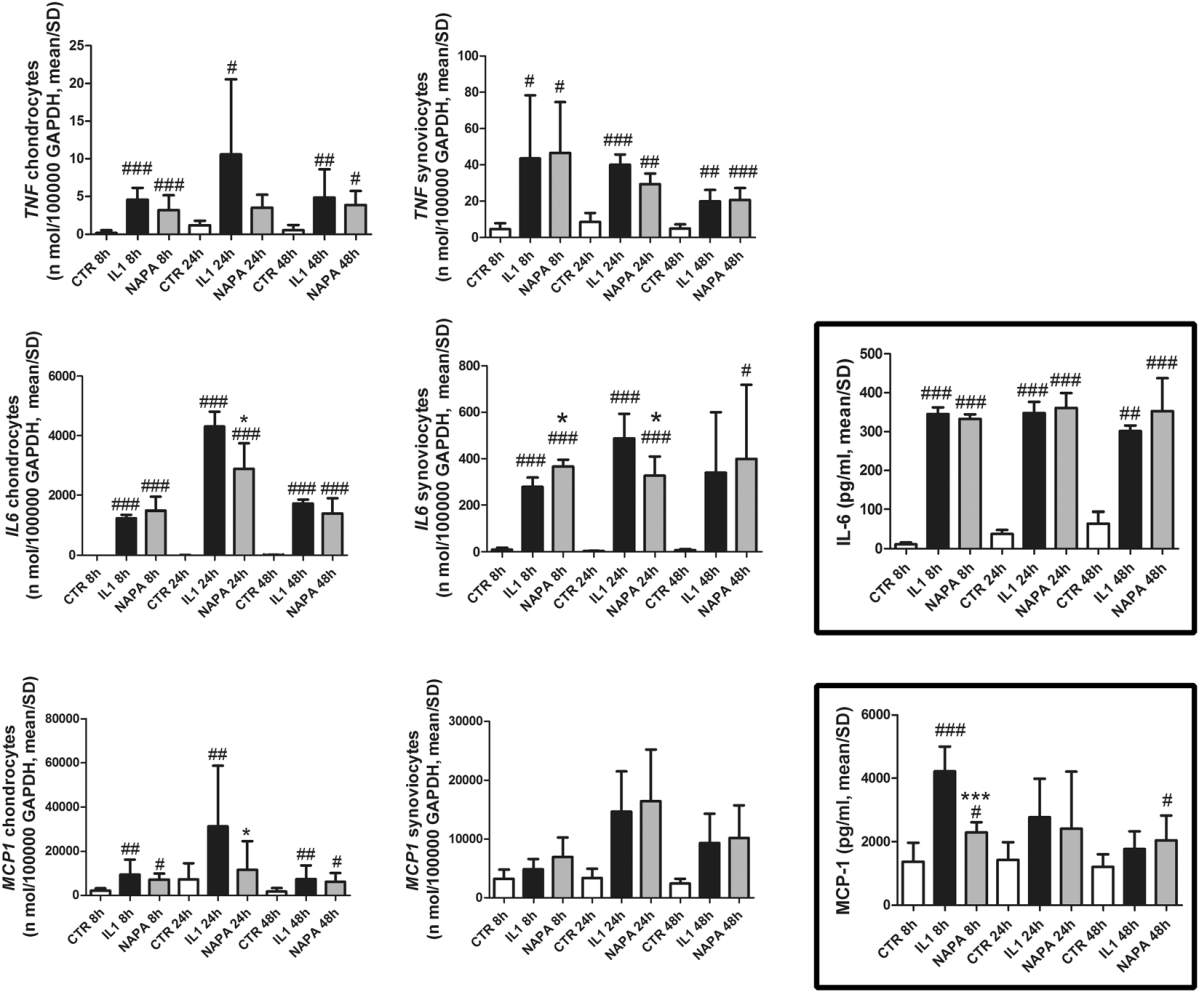
IL-1β strongly upregulates gene expression of pivotal inflammatory genes in chondrocytes, and NAPA counteracts this effect. Left graphs: TNF-α, IL-6 and MCP-1 gene expression in chondrocytes; middle graphs: TNF-α, IL-6 and MCP-1 gene expression in synoviocytes; right framed graphs: protein assessment of IL-6 (above) and MCP-1 (below) in the supernatant of co-cultures [mean ± standard deviation; chondrocytes: n = 8 (4 experiments in duplicate); synoviocytes: n = 4 (2 experiments in duplicate)]. At each time point of NAPA treatment (8, 24 and 48 hours), the means of groups (CTR, IL-1β treated and IL-1β + NAPA treated samples) were compared by ANOVA, followed by Tukey’s post hoc test. Different symbols were used for different comparisons: ^#^P < 0.05; ^##^P < 0.01 and ^###^P < 0.001 were used to score the degree of significance of the differences of either IL-1β treatment or IL-1β + NAPA treatment compared to the control condition and *P < 0.05; **P < 0.01; and ***P < 0.001 for IL-1β treatment compared to the IL-1β + NAPA treatment.

Conversely, the addition of NAPA decreased the chondrocyte expression of most of these genes at 24 hours: ADAMTS5, the pivotal matrix-degrading enzymes in osteoarthritis (Fig. 2) and IL-6 and MCP-1 (Fig. 3). Compared with IL-1β treatment, the addition of NAPA significantly decreased the expression of ADAMTS5 at both 8 and 24 hours (p < 0.01), while MMP13 showed a mild reduction at 24 hours that was not statistically significant. Moreover, NAPA addition significantly reduced IL-6 and MCP-1 (p < 0.05), while TNFα was reduced at 24 hours with NAPA treatment.

Additionally, in synoviocyte cultures, the expression of IL-6 was significantly modulated by NAPA after 24 hours.

**Figure 4:**
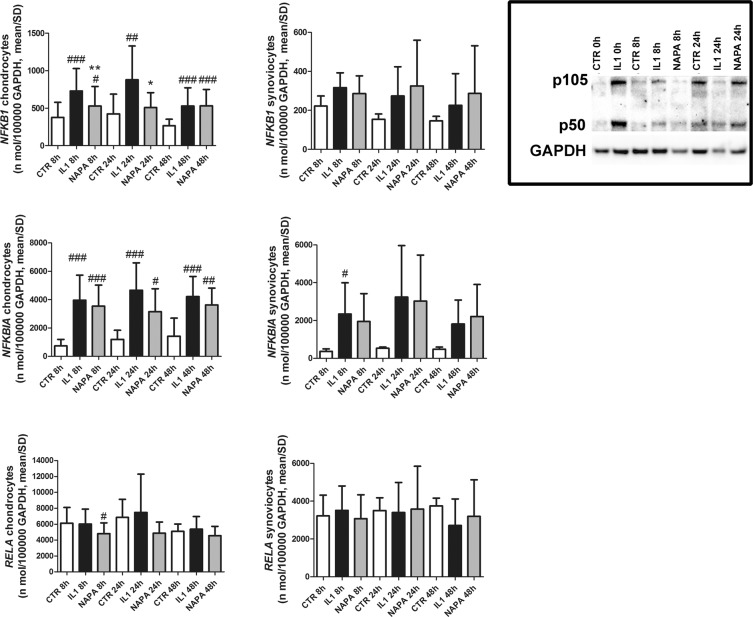
IL-1β upregulates gene expression of some selected NF-κB/Rel proteins in chondrocytes and synoviocytes. NAPA counteracts this effect in chondrocytes. Left graphs: NFKB1 (p105_50), NFKBIA and RELA (p65), gene expression in chondrocytes; middle graphs: NFKB1 (p105_50), NFKBIA and RELA (p65) gene expression in synoviocytes; right framed picture: western blot showing that at 8 hours NAPA is effective in reducing the protein expression of both the p105 precursor and the p50 NF-κB monomer. [mean ± standard deviation; chondrocytes: n = 8 (4 experiments in duplicate); synoviocytes: n = 4 (2 experiments in duplicate)]. At each time point of NAPA treatment (8, 24 and 48 hours), the means of groups (CTR, IL-1β treated and IL-1β + NAPA treated samples) were compared by ANOVA, followed by Tukey’s post hoc test. Different symbols were used for different comparisons: ^#^P < 0.05; ^##^P < 0.01 and ^###^P < 0.001 were used to score the degree of significance of the differences of either IL-1β treatment or IL-1β + NAPA treatment compared to the control condition and *P < 0.05; **P < 0.01; and ***P < 0.001 for IL-1β treatment compared to the IL-1β + NAPA treatment.

To further explore the effects of NAPA in the NF-κB signalling pathway, we investigated the chondrocyte gene expression of three molecules with a role in this pathway: the NF-κB proteins NFKB1 (p105-50) and RelA (p65) and the main inhibitor of this pathway NFKBIA (IκBα). Previous literature has indicated that p105-50 and IκBα are NF-κB targets^27^. NAPA treatment modulated p105-50 at both 8 and 24 hours (p < 0.001 and p < 0.05, respectively, Fig. 4), while RelA was unaffected. NFKBIA gene expression paralleled that of NFKB1 but failed to reach statistical significance. The NAPA-dependent reduction in NFKB1, a known NF-κB target, is consistent with the NF-κB-inhibiting activity of NAPA. On the other hand, RelA, which is not included among the NF-κB-dependent genes, was almost unaffected in both chondrocytes and synoviocytes.

Overall, our data showed a higher expression (in terms of number of molecules relative to those of the housekeeping gene) of these OA-related genes in chondrocytes compared with synoviocytes, together with a higher IL-1β induction and stronger NAPA modulation.

- Protein expression

#### NAPA reduces the levels of key OA effectors in the supernatants of the co-cultures

To confirm that NAPA inhibition of IL-1β-induced transcription was paralleled by effects on the synthesis and release of the same inflammatory markers and matrix degradation effectors, the amounts of these molecules were assessed by enzyme linked assays or BIOPLEX technology for released molecules or by western blot for intracellular proteins.

First, IL-1β was confirmed as a powerful inflammatory trigger at the protein level. To date, we have little evidence about the efficacy of NAPA and only limited knowledge about the specific mechanisms and pathways underlying the anti-inflammatory ability of this innovative drug; therefore, a complete screening of the MMPs was performed. Release of MMP-1, -3, -10, -13, -9, -7, and -12 increased after inflammatory stimulation (Fig. 2, upper right panel and Supplementary Fig. 4). Eight hours of NAPA treatment led to decreased release of MMP-10, MMP-13, MMP-7, although it was not statistically significant.

The pattern of ADAMTS5 was investigated by combining ELISA and western blotting to detect both the intracellular precursor and the released activated enzyme. Western blot analysis was used to assess the ADAMTS5 content of lysates obtained from the high-density chondrocyte cultures. The western blot in the bottom right panel of Fig. 2 shows the 73 kDa protein that corresponds to the active enzyme form yielded by the proteolytic activation of the zymogen. This proteolytic activation results in C-terminal truncation, release of the enzyme from the ECM and alteration of its activity profile^29^. The lower graph represents the values assessed by ELISA in the supernatants collected from one experiment and suggests that NAPA effectively hampers the release of this pivotal matrix degrading enzyme that is induced by inflammatory activation. It is worth emphasizing that in our particular experimental model, the prototypical inflammatory stimulus IL-1β strongly enhances ADAMTS5 expression, thus mostly approaching what was observed in animal models, rather than with short-term stimulation *in vitro*^30^. Despite some specific biochemical differences, ADAMTS5 is much more active than ADAMTS4^29^ and is one among the few genes causatively correlated with OA pathogenesis by functional genomics and surgically induced OA^31^.

With regard to the inflammatory cytokines, the amounts of TNFα (undetectable in the supernatants) or IL-6 (strongly enhanced in the supernatants by IL-1β) were not affected by NAPA treatment. Instead, among the chemokines analysed, after 8 hours of NAPA administration, a strong modulation was observed for MCP-1 (p < 0.001), while milder, but not statistically significant, effects were observed for MIP-1α and MIP-1β (Fig. 3, bottom right panel and Supplementary Fig. 5).

### NAPA modulates IKKα nuclear localization, phosphorylation of Histone 3 (Ser10) and expression of NF-κB/Rel proteins in chondrocytes

#### NAPA reduces IKKα nuclear localization

**Figure 5:**
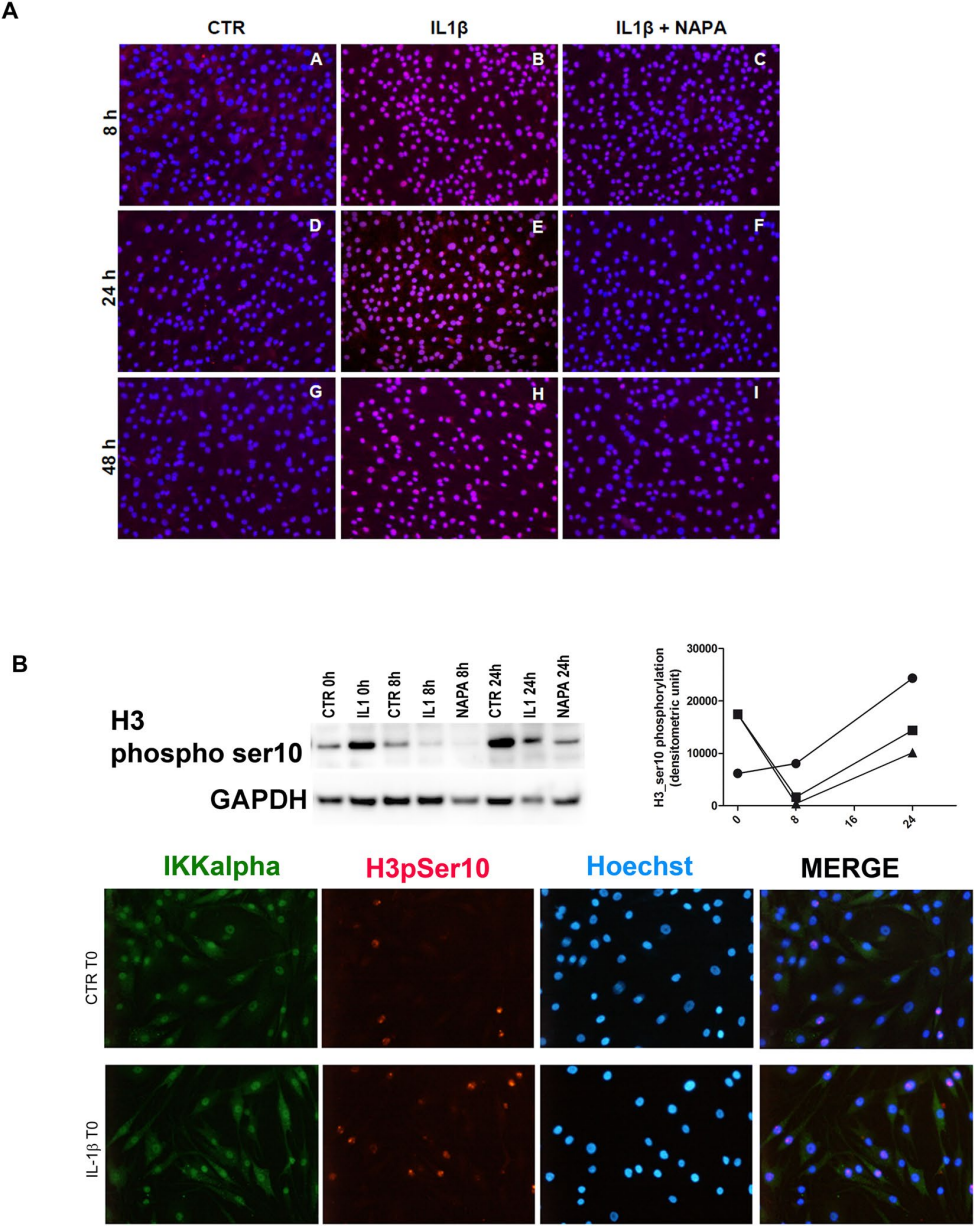
IL-1β increases IKKα nuclear translocation and phosphorylation of serine 10 on histone H3, while NAPA attenuates these signalling events. (**A**) Overlapping signals of nuclear counterstaining (DAPI) and IKKα detected via an Alexa Fluor 555 secondary antibody: the colocalized signals indicate that NAPA addition is effective in reducing nuclear translocation of IKKα. (**B**) Upper pictures: left, western blot of anti-phosphorylated serine 10 of histone H3, along with GAPDH as a loading control and right: densitometric analysis of the signal showing the different pattern of H3pSer10 accumulation in CTR (circle), IL-1β (square) or IL-1β + NAPA (triangle) conditions. Lower images: specificity of the signal obtained with the anti-H3 phosphorylated serine 10: 20x field pictures of chondrocytes grown on coverslips in the bottom of wells at time 0 in control (upper row) or IL-1β stimulated conditions (lower row): Green: IKKα detected with an Alexa Fluor 488 anti-rabbit antibody; red: H3pSer10 signal detected with an Alexa Fluor 555 anti-mouse antibody; blue: nuclear DNA stained with Hoechst 33342 and merged images.

In our experimental model, the immunofluorescence studies confirmed the information available from “The human Protein Atlas”: IKK*α* “is mainly localized to the nucleoplasm and in addition localized to the cytosol and vesicles” (https://www.proteinatlas.org/ENSG00000213341-CHUK/cell). Moreover, IL-1β stimulation led to markedly increased nuclear expression of IKKα at all time points, while NAPA reduced this increase (Fig. 5A), in keeping with previous *in vitro* studies performed with chondrocytic cell lines^5^. The strong nuclear localization is highly suggestive of prevalent chromatin activity of IKKα in this experimental model.

#### NAPA reduces the levels of H3ser10 phosphorylation

Among the IKKα activities performed in the nucleus, a relevant one in the context of the inflammatory stimuli is the phosphorylation of serine 10 of histone H3 (H3Ser10)^32^. Therefore, we performed an analysis of this chromatin modification by western blot, followed by densitometric analysis, and confirmed the specificity of the signal by immunofluorescent staining and positive cell counts.

Western blot analysis allowed us to evaluate the kinetic pattern of the phosphorylation of H3Ser10. At T0, phosphorylated H3Ser10 (H3pSer10) signal appeared evident in the CTR group, in keeping with the basal inflammatory condition of hyperconfluent OA chondrocytes but was markedly increased in the IL-1β group, where the cells had been kept under inflammatory stimulation for one week (Fig. 5B).

It is conceivable that the inflammatory networks in the co-cultures are sustained by both paracrine and autocrine loops, whose weight increases during the experiment, possibly leading, at later times, to biases in evaluating both IL-1β effects and counteracting activity of NAPA. Therefore, the more reliable results are those within 24 hours of medium change. Moreover, previous studies showed that hyperconfluent differentiated chondrocytes present basal activation of p38 MAPK^33^, another kinase responsible for H3pSer10 ^34^. Therefore, the effect of IL-1β needs to be assessed as an enhancement of a basal inflammatory state. The kinetic patterns of H3pSer10 are different in control or IL-1β-stimulated chondrocytes with or without NAPA, indicating a progressive accumulation of the signal in control conditions, while a biphasic pattern was observed following IL-1β stimulation (Fig. 5B, upper western blot pictures), in keeping with literature^34,35^. At time 0, the cells previously treated with IL-1β had a high level of H3pSer10, which markedly decreased at time 8, while NAPA almost completely abolished this signal. Notably, at all time points, NAPA reduces IKKα nuclear translocation. This decrease seems sufficient to affect transcription of the above-mentioned genes, whose expression is indeed significantly reduced by NAPA, mostly at T24, despite, in most cases, a trend of reduction already appreciated at T8. At 24 hours, we observed a second wave of H3Ser10 phosphorylation, and NAPA again reduced this signal. This finding is consistent with the significantly decreased transcription of most target genes at T24.

The analysis of H3pSer10 immunofluorescent staining was carried out in an additional experiment performed with cells plated onto glass coverslips and mostly focused on investigating the pattern of the staining and any differences among cells grown with or without synoviocytes plated onto transwells. Culture on coverslips allowed accurate evaluation by confocal microscopy, but because of differences in cell behaviour when cultured on either plastic or glass surfaces, we matched the functional analysis reported above to the results obtained with western blotting. Nevertheless, the mean percentage of positive cells at T0, after counting many hundreds of cells in several 20x fields, confirmed double the number (mean ± SD) under IL-1β stimulation (Fig. 5B, lower images): CTR T0 4.6 ± 3.2 and IL-1β T0 8.3 ± 5.3.

**Figure 6:**
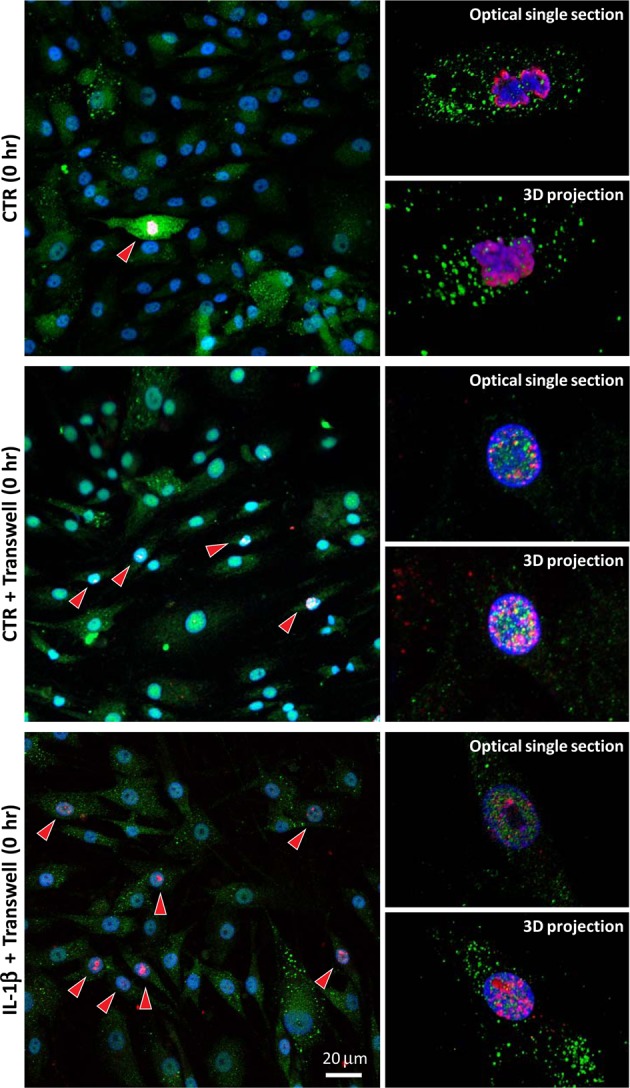
Confocal analysis of the differential subcellular patterns of phosphorylation of serine 10 on histone H3. Upper images: CTR cells at T0 without co-cultured synoviocytes. Chondrocyte culture without transwells showed frequent images of cells undergoing mitosis (left 20x magnification), with strong H3pSer10 signals arranged to stain an outer coat around the supercoiled DNA of the chromosomes (right 60x magnifications: an optical section of the arranged chromosomes in the upper image and the resulting 3D projection of many optical sections in the lower image). Middle images: CTR cells at T0 with co-cultured synoviocytes. Left 20x magnification: chondrocyte culture with transwells showed frequent images of cells in interphase with dotted nuclear H3pSer10 signals, indicating the activation of NF-κB-regulated promoters. Right 60x magnification: an optical section of the nucleus (upper image) and the resulting 3D projection of many optical sections (lower image). Bottom images: IL-1β cells at T0 with co-cultured synoviocytes. Left 20x magnification: IL-1β-stimulated chondrocyte culture with transwells showed more frequent images of cells in interphase with nuclear H3pSer10 signals, indicating increased activation of NF-κB-regulated promoters. Right 60x magnification: an optical section of the nucleus (upper image) and the resulting 3D projection of many optical sections (lower image).

The observation of H3pSer10 immunofluorescent staining in OA chondrocytes either in single culture or co-culture conditions intriguingly revealed mitotic activity only in the absence of synoviocytes (Fig. 6). Bright pictures of chromosomes in prophase were quite frequent when synoviocytes were absent. Conversely, the interaction between the two cell populations seemed to inhibit chondrocyte proliferation and instead sustained the inflammatory state, characterized by a clear nuclear H3pSer10 signal associated with a widespread but mostly nuclear IKKα signal (Figs 5B and 6). This effect strongly points to the added value of exploring the effects of NAPA using the co-culture model, thus obtaining information closer to the microenvironment of an OA joint.

#### NAPA reduces the levels of selected NF-κB/Rel proteins

The western blot shown in Fig. 4 again indicates that the most evident results are obtained at 8 hours and clearly points to IL-1β induction of both the p105 precursor and the p50 NF-κB monomer at T0 and to a marked reduction exerted by NAPA at T8.

## Discussion

State-of-the-art pharmacological approaches to treat OA are focused on the development of safe alternatives for the most common drugs used in the clinic to control their frequent negative side effects. Glucosamine and its analogues seemed very promising, but their clinical use has shown contradictory results so far^20^. Among the GlcN derivatives, NAPA could represent an interesting breakthrough to fight the inflammation associated with OA, but to date, few studies have analysed this molecule that shows chondroprotective roles and inhibitory effects on MMP expression *in vitro*^36^.

An early *in vivo* study indicated that NAPA performed better than GlcN in delaying OA progression in a rabbit model of experimental OA^36^, and this was also confirmed by *in vitro* studies performed with primary rabbit chondrocytes.

More recently, the chondroprotective activities of NAPA were confirmed in a murine surgically induced OA model: the destabilization of medial meniscus (DMM), recognized as the surgical method of choice for reproducing the slow progression of human OA and for identifying the pivotal OA drug targets, as well as testing novel treatments^37^. In this *in vivo* study, NAPA was delivered to the mice 4 weeks after the DMM surgery, thus simulating an early stage of osteoarthritic disease^7,37^. The intra-articular injection of NAPA delayed OA progression and preserved cartilage integrity, reducing fibrillation, cell cluster number and the amount of fibrous tissue, in lateral and medial femoral condyles and in the tibial plateau of knee joints. The immunohistochemical evidence of significantly decreased expression of IKKα in NAPA-treated joints paralleled the decreased expression of ADAMTS5, MMP-10, MMP-13 and therefore was in keeping with our previous findings^38^ about the role of IKKα in OA pathophysiology. This result prompted us to undertake the present *in vitro* study to advance the molecular characterization of the underlying mechanisms and mediators and to confirm the NAPA > IKKα connection. For this purpose, we performed kinetic evaluations that allowed us to explore and correlate signalling events, gene transcription and protein release of many NF-κB target genes with pivotal activity in osteoarthritis^39^.

Based on previous findings^5^, we focused our interest on IKKα, the kinase that is required for the activation of the so-called “non-canonical” pathway^40,41^ but is also able to directly regulate the chromatin structure downstream of the “canonical” pathway and activate transcription of selected genes through phosphorylation of serine 10 of histone H3 (H3Ser10)^40^. Notably, early findings already showed that, compared with IKKβ, IKKα ablation had a broader range of effects on OA chondrocyte physiology^42^, some of which were kinase- and NF-κB- independent and were targeted to the control of ECM remodelling via tuning of MMP-13 activity^38^ without marked effects on MMP-13 gene and protein expression^43^.

The experimental co-culture model exploited the use of primary human chondrocytes and synoviocytes and was selected to better mimic the environment of an OA joint *in vitro*, with the crosstalk between cartilage and synovium^44^ and therefore was a suitable model for translating the findings to human pathology, as well as disclosing any “site specific” effects. Before any treatment, chondrocytes were allowed to reach high density to recover a differentiated chondrogenic phenotype^24^. To reproduce an inflamed OA joint, the co-cultures were long-term stimulated with IL-1β, a recognized OA target validated in DMM^31^. In these experimental settings, cells from either healthy or OA donors behaved in the same way, pointing to the strength of our experimental co-culture model and its reliability in reproducing an OA environment, independent of the healthy or OA origin of the joint cells. Therefore, we pooled the findings to increase the sample sizes.

To gain insight into the signalling events, we evaluated the trend of H3Ser10 phosphorylation by western blot and confirmed specificity by immunofluorescence, selecting the early experimental time points (0, 8 and 24 hours). Moreover, to assess synoviocyte contribution to the inflammatory environment, we also performed immunofluorescence experiments to compare the phosphorylated H3Ser10 pattern of plain chondrocytes with that of chondrocytes in co-culture with synoviocytes and found that synoviocytes enhance an “inflammatory” H3pSer10 pattern. Indeed, as previously reviewed^45^, phosphorylated H3Ser10 antibody brilliantly marks chromosome condensation in cell division, while in interphase, chromatin relaxation required to allow transcription of NF-κB regulated promoters. Several different kinases may intervene in phosphorylating H3Ser10^45^, but only IKKα “functions in the nucleus to activate the expression of NF-κB responsive genes after stimulation with cytokines”. In these conditions, IKKα is recruited to NF-κB-responsive promoters, and there confers a permissive arrangement to the chromatin by mediating H3Ser10 phosphorylation and subsequent acetylation of specific residues^46^. Previous work has described the latency between H3Ser10 phosphorylation and transcription of selected genes^34^: H3Ser10 phosphorylation is, in most cases, a phasic event that upon inflammatory cytokine stimulation tags some NF-κB-regulated promoters and shortly precedes the start of transcription^34^ of genes encoding for inflammatory mediators, thus leading to an “autocrine” enhancement of NF-κB signalling.

However, the cells have developed molecular mechanisms whereby they downregulate inflammation. Our findings indeed highlighted an overall irregular trend in the gene expression of most inflammatory markers under IL-1β stimulation, which peaked at T24 but decreased at 48 hours, possibly because of negative feedback loops^35^ or receptor desensitization^47^. Notably, in these experimental settings at 24 hours, NAPA significantly decreased the gene expression of ADAMTS5, IL-6 and MCP-1 in one or both cell types compared to the IL-1β stimulated samples. NFKB1 (p105-p50) and IL-6 are important NF-κB target genes^27^ whose reduction points to effective inhibition of the NF-κB signalling pathway exerted by NAPA at the early time points.

Despite the difficulty in exactly defining the timing of gene transcription after chromatin activation^26,34^, the aforementioned results could be related, at least partially, to what was observed in the western blot analysis of H3Ser10 phosphorylation in the chondrocytes. The kinetics of H3Ser10 phosphorylation show a different trend among CTR cells and cells stimulated with IL-1β, either with or without NAPA. At time 0, the cells previously treated with IL-1β had a high level of H3pSer10, which markedly decreased at time 8, while NAPA almost completely abolished this signal. It is likely that this effect is dependent on the ability of NAPA to reduce IKKα nuclear translocation, as shown by immunofluorescent experiments. This ability seems sufficient to affect transcription of the above-mentioned genes, whose expression is indeed significantly reduced, mostly at T24, despite, in most cases, a trend of reduction that is already appreciated at T8. At 24 hours, we observed a second wave of H3Ser10 phosphorylation, and NAPA again reduced this signal. The early effects of NAPA in reducing transcription at T8 are possibly quickly reflected in translation since our protein release analysis indicated a significant reduction by NAPA of some inflammatory or catabolic effectors, mostly at early time points (T8). However, it is also conceivable that the early effects of NAPA on protein production might be dependent on the medium change performed at the time of NAPA delivery, while later accumulation phenomena might mask these effects. Given our experimental settings, in all groups, and for almost all the quantified molecules, we appreciated a time-dependent progressively increasing release as proof of the accumulation of the cellular products that hampered the perception of NAPA effects at later time points. Moreover, accumulating evidence indicates that despite the pivotal role of the NF-κB signalling pathway in OA, involvement of other signalling pathways may occur downstream in chondrocytes in response to stimulation with IL-1β^48^. Interaction between the NF-κB and the Notch pathway has been recently reviewed^49^, and perhaps the differential ability of NAPA to counteract transcription and translation of a given gene may depend on the prevalent or accessory role of NF-κB in that process. Notably, MCP-1 was significantly reduced in the presence of NAPA at T8. Milder, not statistically significant effects were observed for RANTES, MIP-1α and β and metalloproteases, such as MMP13 and MMP7.

The role of chemokines in OA has been recently reviewed and disclosed based on cumulative evidence derived from both findings obtained in patients and evidence obtained in animal models of OA. The severity of human OA, as assessed by the mean of the radiological score, has been found to be correlated with the levels in synovial fluid of selected chemokines that include those analysed in our study, i.e., the CCR2 ligand (CCL2/MCP-1) and CCR5 ligands (CCL3/MIP-1*α*, CCL4/MIP-1*β*, and CCL5/RANTES)^50^. Particularly, CCL2/MCP-1 is the major chemokine active in macrophage recruitment and therefore plays a role in the activation of innate immunity, now recognized to contribute to OA pathogenesis. The pivotal role of this chemokine in OA development is confirmed by an early increase in CCL2 transcription that follows two surgical OA models: the murine DMM^51^ and the rat meniscal tear models^52^. CCL2/MCP-1 serum levels were correlated with both pain scores^53^ and with radiographic progression of OA^54^. A recent report that combined human findings *ex vivo* with results from DMM mice further confirmed a key role of MCP-1 in mediating monocyte recruitment, inflammation and cartilage destruction^55^ in osteoarthritis.

A relevant effect of NAPA is the modulation of ADAMTS5, the foremost cartilage ECM-degrading enzyme responsible for aggrecan removal in the early phase of the disease^56^. This pivotal role is confirmed by the effectiveness of inhibiting OA progression by a treatment of this catabolic enzyme by small interfering RNA^56^. Notably, ADAMTS5 is an OA drug target validated with the DMM model^31^, and indeed, its antibody-mediated neutralization has proven able to inhibit OA progression in a DMM model^57^. The ability of NAPA to inhibit transcription and release of multiple inflammatory mediators and catabolic enzymes in human primary joint cells further points to this drug as a suitable candidate for the therapeutic treatment of OA. A careful refinement of the delivery schedule of this drug or slow releasing strategies would be needed for the long-term maintenance of its anti-inflammatory and anti-catabolic activity.

The pattern of transcription upon IL-1β stimulation, with or without NAPA, was similar in both cell types in some cases (such as for IL-6), but overall, we observed that chondrocytes had a higher IL-1β induction and a stronger NAPA modulation, thus pointing at “site specific” effects.

We previously described the differential involvement of cartilage and synovium in the pathogenesis of either osteoarthritis or inflammatory arthritis, based on the co-localization of IL-1β, TNFα and iNOS^58^, suggesting NF-κB activation in either the cartilage or the synovium since the three markers are direct NF-κB targets^27^. The primary role of cartilage in driving joint derangement in OA has also been recently confirmed^59^. On the other hand, as was recently reviewed^60^, histopathological studies have documented changes of progressively increasing severity in synovial samples derived from patients with different OA grades compared with normal synovium, which consists of a thin lining layer an a loose connective tissue subintimal layer. Changes range from synovial lining hyperplasia, villous hyperplasia, fibrosis and infiltration of perivascular mononuclear cells that give rise to the infiltrating macrophages that may be found in both the subintimal layer and in the perivascular infiltrates, where they are also able to recruit T and B lymphocytes.

Thus, in OA synovial samples, both fibroblast-like and macrophage cells are present, with a higher percentage of the latter in moderate- compared to low-grade synovitis. The behaviour of the cells derived from the synovial samples changes with regard to the composition of prevalent fibroblasts or mixed fibroblasts/macrophages^61^. In keeping with their higher prevalence in more advanced stages of the disease, synovial macrophages indeed show a higher level of inflammation, with stronger release of chemokines and alarmins compared to the synovial fibroblasts.

In the present study, we used human fibroblast-like synoviocytes of both normal and OA origin (https://www.cellapplications.com/human-fibroblast-synoviocytes-hfls). Therefore, we have been working with cells that are closer to the cells of the lining layer, the only synovial component present in the early stages of the disease that are just a bystander of the damage that is initially triggered in the cartilage and then involves the synovium^44^.

## Supplementary information

Supplementary Material

## Data Availability

The data reported in this study are available from the corresponding author.

## References

[1] Pers YM, Ruiz M, Noel D, Jorgensen C (2015). Mesenchymal stem cells for the management of inflammation in osteoarthritis: state of the art and perspectives. Osteoarthritis Cartilage.

[2] Attur MG, Dave M, Akamatsu M, Katoh M, Amin AR (2002). Osteoarthritis or osteoarthrosis: the definition of inflammation becomes a semantic issue in the genomic era of molecular medicine. Osteoarthritis Cartilage.

[3] Goldring SR, Goldring MB (2016). Changes in the osteochondral unit during osteoarthritis: structure, function and cartilage-bone crosstalk. Nat Rev Rheumatol.

[4] Martel-Pelletier J (2016). Osteoarthritis. Nat Rev Dis Primers.

[5] Scotto d’Abusco A, Politi L, Giordano C, Scandurra R (2010). A peptidyl-glucosamine derivative affects IKKalpha kinase activity in human chondrocytes. Arthritis Res Ther.

[6] Olivotto E, Otero M, Marcu KB, Goldring MB (2015). Pathophysiology of osteoarthritis: canonical NF-kappaB/IKKbeta-dependent and kinase-independent effects of IKKalpha in cartilage degradation and chondrocyte differentiation. RMD Open.

[7] Veronesi, F. *et al*. Chondroprotective activity of N-acetyl phenylalanine glucosamine derivative on knee joint structure and inflammation in a murine model of osteoarthritis. *Osteoarthritis Cartilage* **25**, 589–599, doi:S1063-4584(16)30363-6 (2017). 10.1016/j.joca.2016.10.021 27836674

[8] Goldring, M. B. *et al*. Roles of inflammatory and anabolic cytokines in cartilage metabolism: signals and multiple effectors converge upon MMP-13 regulation in osteoarthritis. *Eur Cell Mater* **21**, 202–220, doi:vol021a16 (2011). 10.22203/ecm.v021a16 PMC3937960 21351054

[9] Migliore A, Procopio S (2015). Effectiveness and utility of hyaluronic acid in osteoarthritis. Clin Cases Miner Bone Metab.

[10] Dougados M (2001). Evaluation of the structure-modifying effects of diacerein in hip osteoarthritis: ECHODIAH, a three-year, placebo-controlled trial. Evaluation of the Chondromodulating Effect of Diacerein in OA of the Hip. Arthritis Rheum.

[11] Schulze-Tanzil, G., Mobasheri, A., Sendzik, J., John, T. & Shakibaei, M. Effects of curcumin (diferuloylmethane) on nuclear factor kappaB signaling in interleukin-1beta-stimulated chondrocytes. *Ann N Y Acad Sci* **1030**, 578–586, doi:1030/1/578 (2004). 10.1196/annals.1329.067 15659840

[12] Shikhman AR, Kuhn K, Alaaeddine N, Lotz M (2001). N-acetylglucosamine prevents IL-1 beta-mediated activation of human chondrocytes. J Immunol.

[13] Scotto d’Abusco, A. *et al*. Glucosamine affects intracellular signalling through inhibition of mitogen-activated protein kinase phosphorylation in human chondrocytes. *Arthritis Res Ther* **9**, R104, doi:ar2307 (2007). 10.1186/ar2307 PMC2212570 17925024

[14] Scotto d’Abusco, A. *et al*. Glucosamine and its N-acetyl-phenylalanine derivative prevent TNF-alpha-induced transcriptional activation in human chondrocytes. *Clin Exp Rheumatol* **25**, 847–852, doi:2214 (2007). 18173918

[15] Chan, P. S., Caron, J. P., Rosa, G. J. & Orth, M. W. Glucosamine and chondroitin sulfate regulate gene expression and synthesis of nitric oxide and prostaglandin E(2) in articular cartilage explants. *Osteoarthritis Cartilage* **13**, 387–394, doi:S1063-4584(05)00019-1 2005). 10.1016/j.joca.2005.01.003 15882562

[16] FENTON J. I., CHLEBEK-BROWN K. A., CARON J. P., ORTH M. W. (2010). Effect of glucosamine on interleukin-1-conditioned articular cartilage. Equine Veterinary Journal.

[17] Uitterlinden, E. J. *et al*. Glucosamine decreases expression of anabolic and catabolic genes in human osteoarthritic cartilage explants. *Osteoarthritis Cartilage* **14**, 250–257, doi:S1063-4584(05)00284-0 (2006). 10.1016/j.joca.2005.10.001 16300972

[18] Dodge, G. R. & Jimenez, S. A. Glucosamine sulfate modulates the levels of aggrecan and matrix metalloproteinase-3 synthesized by cultured human osteoarthritis articular chondrocytes. *Osteoarthritis Cartilage* **11**, 424–432, doi:S1063458403000529 (2003). 10.1016/s1063-4584(03)00052-9 12801482

[19] de Mattei, M. *et al*. High doses of glucosamine-HCl have detrimental effects on bovine articular cartilage explants cultured *in vitro*. *Osteoarthritis Cartilage* **10**, 816–825, doi:S1063458402908344 (2002). 10.1053/joca.2002.0834 12359168

[20] Rozendaal RM (2009). Effect of glucosamine sulphate on joint space narrowing, pain and function in patients with hip osteoarthritis; subgroup analyses of a randomized controlled trial. Osteoarthritis Cartilage.

[21] Sawitzke AD (2010). Clinical efficacy and safety of glucosamine, chondroitin sulphate, their combination, celecoxib or placebo taken to treat osteoarthritis of the knee: 2-year results from GAIT. Ann Rheum Dis.

[22] Wilkens P, Scheel IB, Grundnes O, Hellum C, Storheim K (2010). Effect of glucosamine on pain-related disability in patients with chronic low back pain and degenerative lumbar osteoarthritis: a randomized controlled trial. JAMA.

[23] Scanzello CR (2011). Synovial inflammation in patients undergoing arthroscopic meniscectomy: molecular characterization and relationship to symptoms. Arthritis Rheum.

[24] Ulivi V, Giannoni P, Gentili C, Cancedda R, Descalzi F (2008). p38/NF-kB-dependent expression of COX-2 during differentiation and inflammatory response of chondrocytes. J Cell Biochem.

[25] Schmittgen TD, Livak KJ (2008). Analyzing real-time PCR data by the comparative C(T) method. Nat Protoc.

[26] Assirelli E (2014). Human osteoarthritic cartilage shows reduced *in vivo* expression of IL-4, a chondroprotective cytokine that differentially modulates IL-1beta-stimulated production of chemokines and matrix-degrading enzymes *in vitro*. PLoS One.

[27] Oeckinghaus A, Ghosh S (2009). The NF-kappaB family of transcription factors and its regulation. Cold Spring Harb Perspect Biol.

[28] Murakami S, Lefebvre V, de Crombrugghe B (2000). Potent inhibition of the master chondrogenic factor Sox9 gene by interleukin-1 and tumor necrosis factor-alpha. J Biol Chem.

[29] Verma P, Dalal K (2011). ADAMTS-4 and ADAMTS-5: key enzymes in osteoarthritis. J Cell Biochem.

[30] Fosang, A. J., Rogerson, F. M., East, C. J. & Stanton, H. ADAMTS-5: the story so far. *Eur Cell Mater* **15**, 11–26, doi:vol015a02 (2008). 10.22203/ecm.v015a02 18247274

[31] Glasson SS (2007). *In vivo* osteoarthritis target validation utilizing genetically-modified mice. Curr Drug Targets.

[32] Anest V (2003). A nucleosomal function for IkappaB kinase-alpha in NF-kappaB-dependent gene expression. Nature.

[33] Ulivi V (2006). A common pathway in differentiation and inflammation: p38 mediates expression of the acute phase SIP24 iron binding lipocalin in chondrocytes. J Cell Physiol.

[34] Saccani S, Pantano S, Natoli G (2002). p38-Dependent marking of inflammatory genes for increased NF-kappa B recruitment. Nat Immunol.

[35] Chen BC, Wu WT, Ho FM, Lin WW (2002). Inhibition of interleukin-1beta -induced NF-kappa B activation by calcium/calmodulin-dependent protein kinase kinase occurs through Akt activation associated with interleukin-1 receptor-associated kinase phosphorylation and uncoupling of MyD88. J Biol Chem.

[36] Scotto d’Abusco A (2008). Effects of intra-articular administration of glucosamine and a peptidyl-glucosamine derivative in a rabbit model of experimental osteoarthritis: a pilot study. Rheumatol Int.

[37] Glasson, S. S., Blanchet, T. J. & Morris, E. A. The surgical destabilization of the medial meniscus (DMM) model of osteoarthritis in the 129/SvEv mouse. *Osteoarthritis Cartilage* **15**, 1061–1069, doi:S1063-4584(07)00110-0 (2007). 10.1016/j.joca.2007.03.006 17470400

[38] Olivotto E (2013). IKKalpha/CHUK regulates extracellular matrix remodeling independent of its kinase activity to facilitate articular chondrocyte differentiation. PLoS One.

[39] Marcu, K. B., Otero, M., Olivotto, E., Borzi, R. M. & Goldring, M. B. NF-kappaB signaling: multiple angles to target OA. *Curr Drug Targets* **11**, 599–613, doi:CDT-Aigner HT-9 (2010). 10.2174/138945010791011938 PMC3076145 20199390

[40] Perkins, N. D. Integrating cell-signalling pathways with NF-kappaB and IKK function. *Nat Rev Mol Cell Biol* **8**, 49–62, doi:nrm2083 (2007). 10.1038/nrm2083 17183360

[41] Sun SC (2011). Non-canonical NF-kappaB signaling pathway. Cell Res.

[42] Olivotto E (2008). Differential requirements for IKKalpha and IKKbeta in the differentiation of primary human osteoarthritic chondrocytes. Arthritis Rheum.

[43] Culley KL (2019). Inducible knockout of CHUK/IKKalpha in adult chondrocytes reduces progression of cartilage degradation in a surgical model of osteoarthritis. Sci Rep.

[44] Nefla M, Holzinger D, Berenbaum F, Jacques C (2016). The danger from within: alarmins in arthritis. Nat Rev Rheumatol.

[45] Prigent C, Dimitrov S (2003). Phosphorylation of serine 10 in histone H3, what for?. J Cell Sci.

[46] Yamamoto Y, Verma UN, Prajapati S, Kwak YT, Gaynor RB (2003). Histone H3 phosphorylation by IKK-alpha is critical for cytokine-induced gene expression. Nature.

[47] McKean DJ, Huntoon C, Bell M (1994). Ligand-induced desensitization of interleukin 1 receptor-initiated intracellular signaling events in T helper lymphocytes. J Exp Med.

[48] Ottaviani S (2010). Hes1, a new target for interleukin 1beta in chondrocytes. Ann Rheum Dis.

[49] Saito T, Tanaka S (2017). Molecular mechanisms underlying osteoarthritis development: Notch and NF-kappaB. Arthritis Res Ther.

[50] Scanzello CR (2017). Chemokines and inflammation in osteoarthritis: Insights from patients and animal models. J Orthop Res.

[51] Miotla Zarebska, J. *et al*. CCL2 and CCR2 regulate pain-related behaviour and early gene expression in post-traumatic murine osteoarthritis but contribute little to chondropathy. *Osteoarthritis Cartilage* **25**, 406–412, doi:S1063-4584(16)30320-X (2017). 10.1016/j.joca.2016.10.008 PMC5358501 27746376

[52] Wei T (2010). Analysis of early changes in the articular cartilage transcriptisome in the rat meniscal tear model of osteoarthritis: pathway comparisons with the rat anterior cruciate transection model and with human osteoarthritic cartilage. Osteoarthritis Cartilage.

[53] Li L, Jiang BE (2015). Serum and synovial fluid chemokine ligand 2/monocyte chemoattractant protein 1 concentrations correlates with symptomatic severity in patients with knee osteoarthritis. Ann Clin Biochem.

[54] Longobardi, L. *et al*. Associations between the chemokine biomarker CCL2 and knee osteoarthritis outcomes: the Johnston County Osteoarthritis Project. *Osteoarthritis Cartilage* **26**, 1257–1261, doi:S1063-4584(18)31216-0 (2018). 10.1016/j.joca.2018.04.012 PMC6098742 29723633

[55] Raghu H (2017). CCL2/CCR2, but not CCL5/CCR5, mediates monocyte recruitment, inflammation and cartilage destruction in osteoarthritis. Ann Rheum Dis.

[56] Hoshi H (2017). Effect of inhibiting MMP13 and ADAMTS5 by intra-articular injection of small interfering RNA in a surgically induced osteoarthritis model of mice. Cell Tissue Res.

[57] Miller RE, Tran PB, Ishihara S, Larkin J, Malfait AM (2016). Therapeutic effects of an anti-ADAMTS-5 antibody on joint damage and mechanical allodynia in a murine model of osteoarthritis. Osteoarthritis Cartilage.

[58] Melchiorri, C. *et al*. Enhanced and coordinated *in vivo* expression of inflammatory cytokines and nitric oxide synthase by chondrocytes from patients with osteoarthritis. *Arthritis Rheum* **41**, 2165–2174, doi:10.1002/1529-0131(199812)41:12<2165::AID-ART11>3.0.CO;2-O (1998). 10.1002/1529-0131(199812)41:12<2165::AID-ART11>3.0.CO;2-O 9870873

[59] Pearson MJ (2017). IL-6 secretion in osteoarthritis patients is mediated by chondrocyte-synovial fibroblast cross-talk and is enhanced by obesity. Sci Rep.

[60] Scanzello CR, Goldring SR (2012). The role of synovitis in osteoarthritis pathogenesis. Bone.

[61] Manferdini C (2016). From osteoarthritic synovium to synovial-derived cells characterization: synovial macrophages are key effector cells. Arthritis Res Ther.

