# Supplementary material for: The N-Acetyl Phenylalanine Glucosamine Derivative Attenuates the Inflammatory/Catabolic Environment in a Chondrocyte-Synoviocyte Co-Culture System
Source: Sci Rep. 2019 Sep 19;9:13603. doi: 10.1038/s41598-019-49188-9 (PMC6753094; doi:10.1038/s41598-019-49188-9)
Supplement: Supplementary file 1 — Supplementary Material [file 41598_2019_49188_MOESM1_ESM.pdf]

Supplementary Material

THE N-ACETYL PHENYLALANINE GLUCOSAMINE DERIVATIVE ATTENUATES THE INFLAMMATORY/CATABOLIC ENVIRONMENT IN A CHONDROCYTE-SYNOVIOCYTE CO-CULTURE SYSTEM

Stefania Pagani, Manuela Minguzzi, Laura Sicuro, Francesca Veronesi, Spartaco Santi, Anna Scotto D'Abusco, Milena Fini and Rosa Maria Borzì

S1

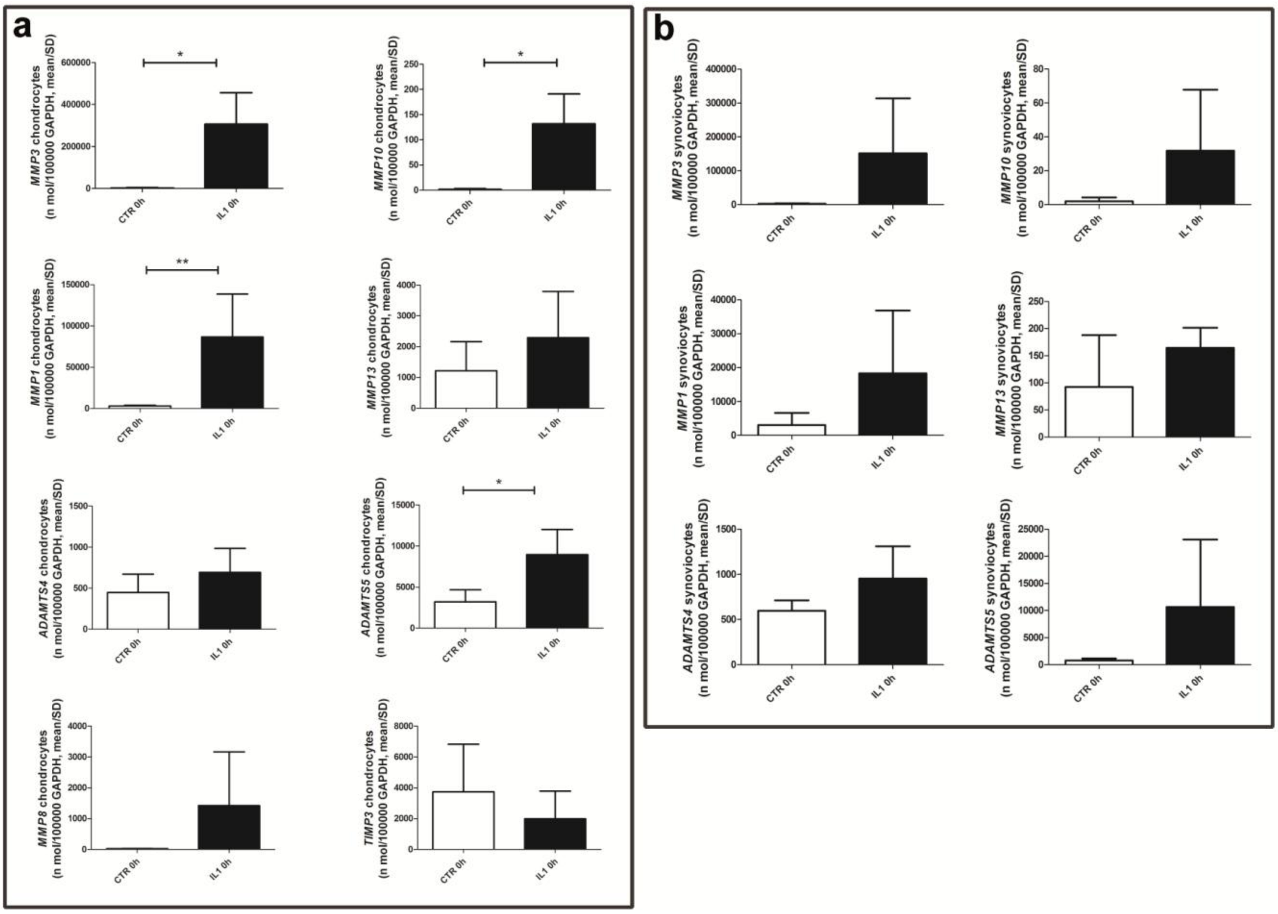

Supplementary Figure 1 – Long term (1 week) IL-1β stimulation upregulates gene expression of pivotal catabolic genes in chondrocytes (left group) and synoviocytes (right group). IL-1β significantly increased chondrocyte gene expression of MMP-3, MMP-10, MMP-1 and ADAMTS-5. Data are represented as mean ± standard deviation; chondrocytes: n = 8 (4 experiments with duplicate); synoviocytes: n = 4 (2 experiments with duplicate)**.** Means of groups were compared with Two tailed Student's t test and considered significant when $P < 0.05$, with *$P < 0.05$ and **$P < 0.01$.

S2

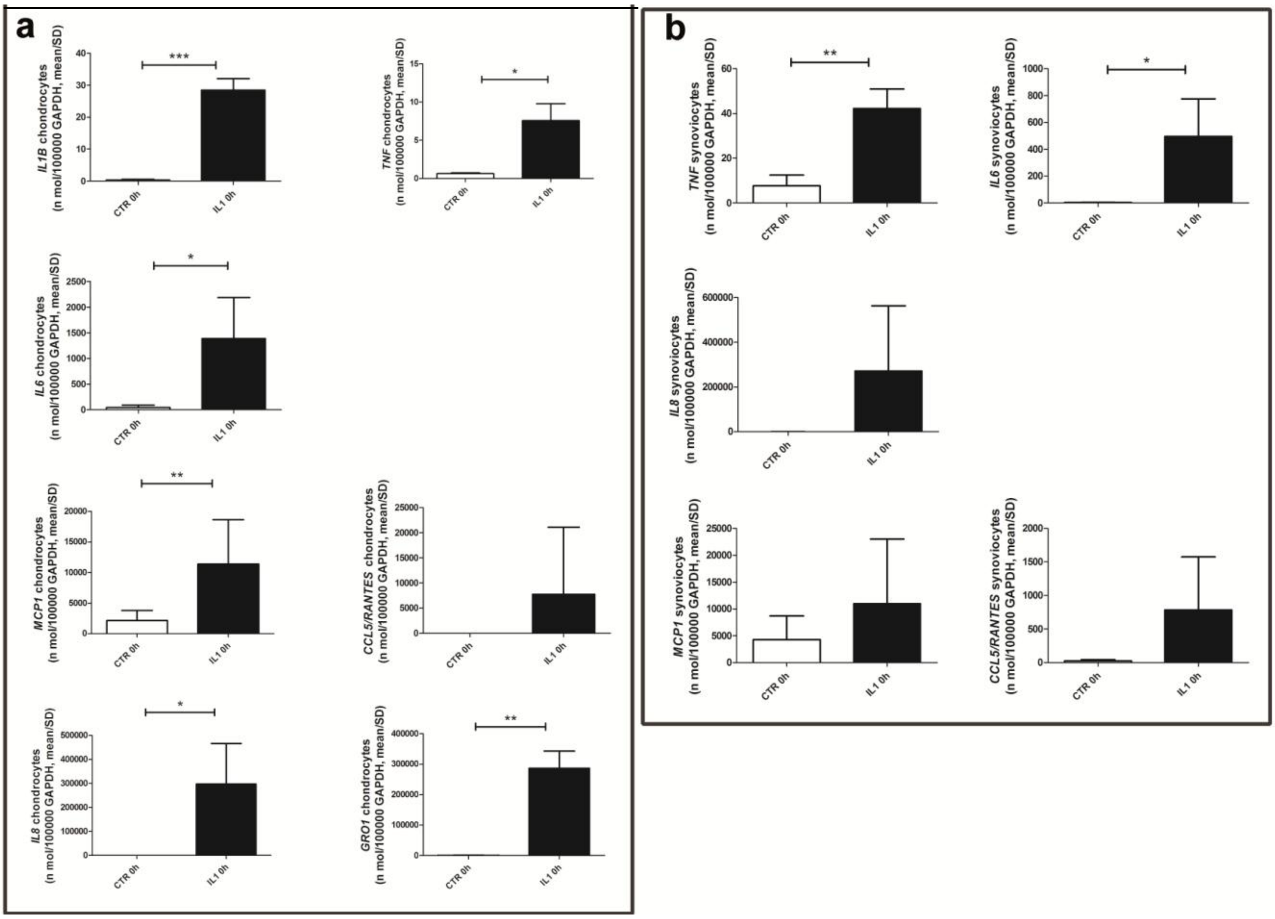

Supplementary Figure 2 – Long term (1 week) IL-1β stimulation upregulates gene expression of pivotal inflammatory genes in chondrocytes (left group: IL-1β, TNFα, IL-6, MCP-1, IL-8 and GROα) and synoviocytes (right group: TNFα and IL-6). Data are represented as mean ± standard deviation; chondrocytes: n = 8 (4 experiments with duplicate); synoviocytes: n = 4 (2 experiments with duplicate)**.** Means of groups were compared with Two tailed Student's t test and considered significant when $P < 0.05$, with *$P < 0.05$; **$P < 0.01$; ***$P < 0.001$.

S3

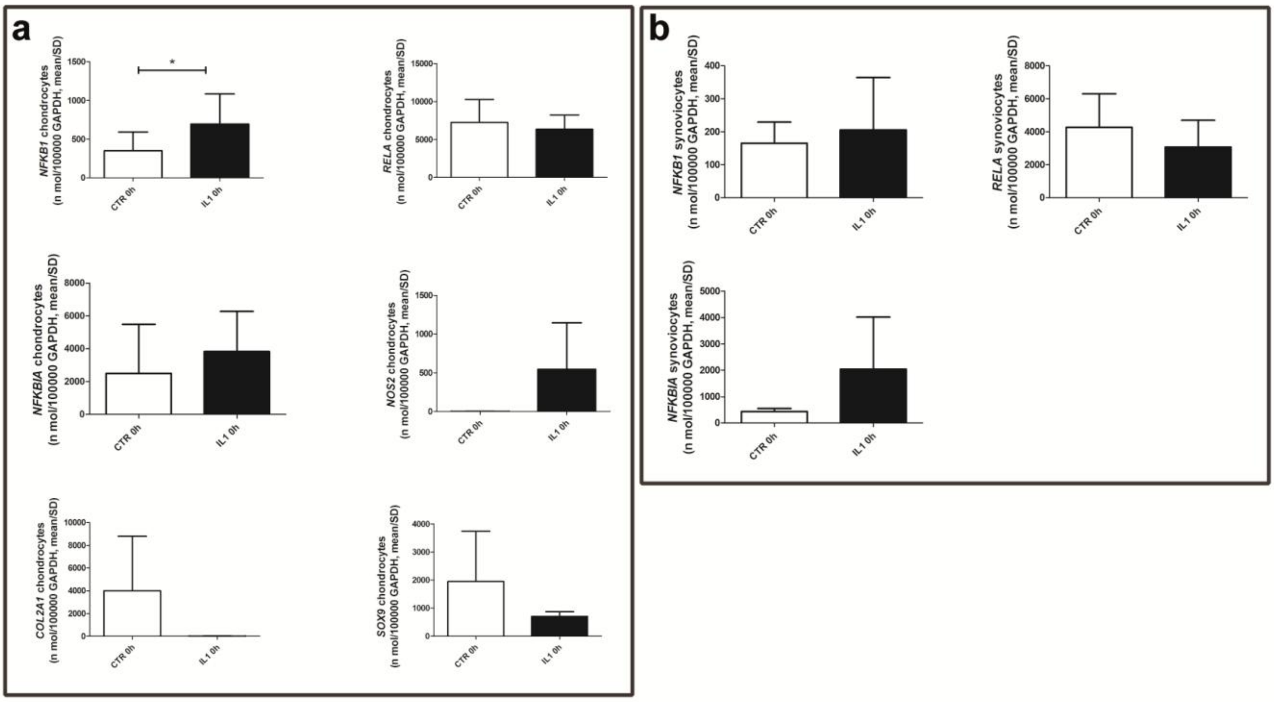

Supplementary Figure 3 – Long term (1 week) IL-1β stimulation upregulates gene expression of selected NF-κB/Rel in chondrocytes (left group: NFKB1) but not in synoviocytes (right group). Data are represented as mean ± standard deviation; chondrocytes: n = 8 (4 experiments with duplicate); synoviocytes: n = 4 (2 experiments with duplicate)**.** Means of groups were compared with Two tailed Student's t test and considered significant when $P < 0.05$, with $*P < 0.05$.

S4

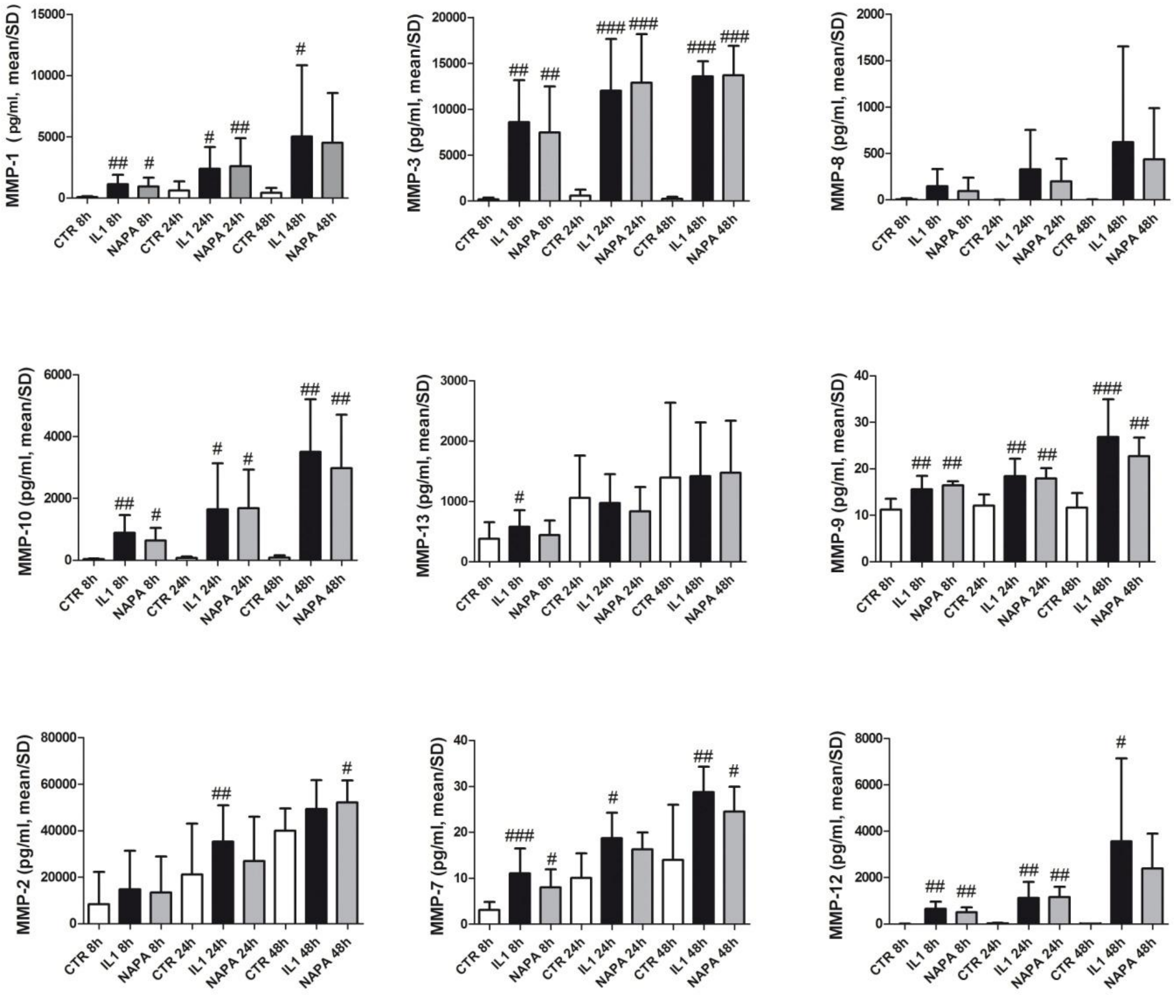

Supplementary Figure 4 – IL-1β upregulates protein release of pivotal catabolic enzymes. NAPA treatment slightly reduced the amount of released MMP-13 and MMP-7 at 8 hours. Data are represented as mean ± standard deviation of 3 different experiments with duplicate samples. At each time point of NAPA treatment (8, 24 and 48 hours), means of groups (CTR, IL-1β treated and IL-1β+NAPA treated samples) were compared by ANOVA analysis, followed by Tukey post hoc test. #P < 0.05; ##P < 0.01 and ###P < 0.001 were used to score the degree of significance of the differences of either IL-1β treatment or IL-1β+NAPA treatment compared the control condition.

S5

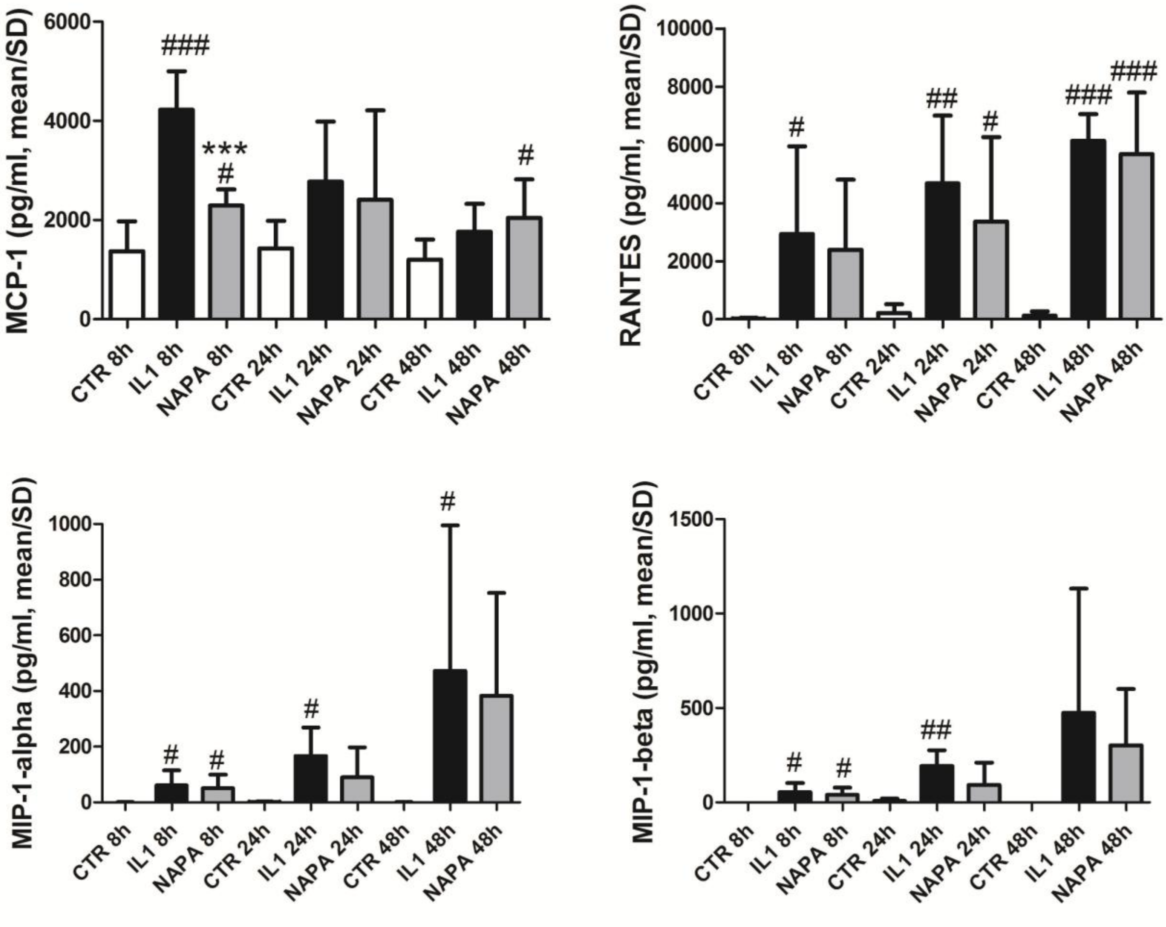

Supplementary Figure 5 – IL-1β up-regulates protein release of pivotal chemokines in OA. NAPA treatment proved to significantly reduce the amount of released MCP-1 at 8 hours. Data are represented as mean ± standard deviation of 3 different experiments with duplicate samples. At each time point of NAPA treatment (8, 24 and 48 hours), means of groups (CTR, IL-1β treated and IL-1β+NAPA treated samples) were compared by ANOVA analysis, followed by Tukey post hoc test. Different symbols were used for different comparisons: #P < 0.05; ##P < 0.01 and ###P < 0.001 were used to score the degree of significance of the differences of either IL-1β treatment or IL-1β+NAPA treatment compared the control condition and *P < 0.05; **P < 0.01; ***P < 0.001 for IL-1β treatment compared to the IL-1β+NAPA treatment.

S6

A

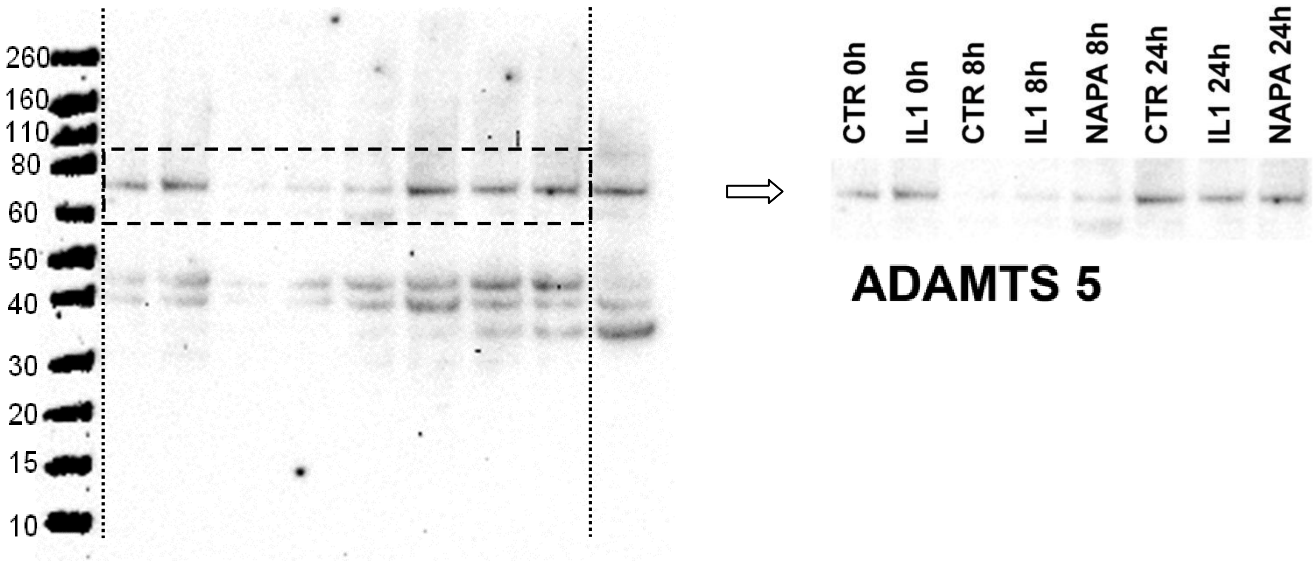
260
160
110
80
60
50
40
30
20
15
10
CTR 0h
IL1 0h
CTR 8h
IL1 8h
NAPA 8h
CTR 24h
IL1 24h
NAPA 24h
ADAMTS 5

B

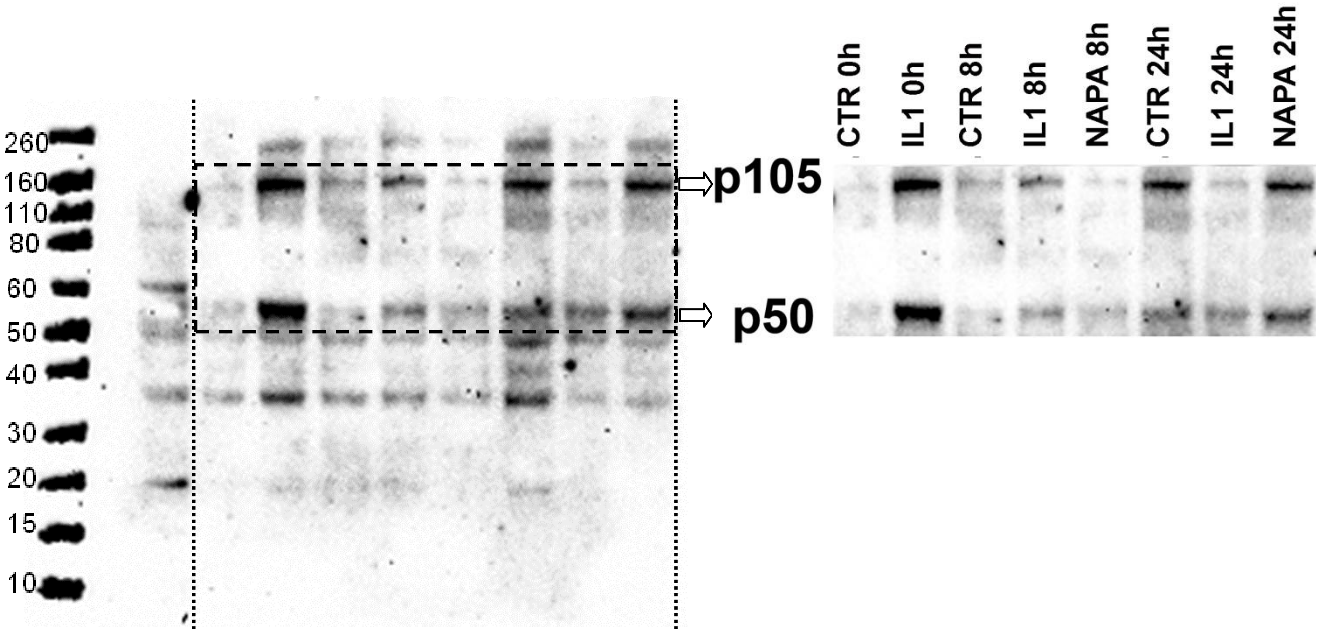
260
160
110
80
60
50
40
30
20
15
10
p105
p50
CTR 0h
IL1 0h
CTR 8h
IL1 8h
NAPA 8h
CTR 24h
IL1 24h
NAPA 24h

C

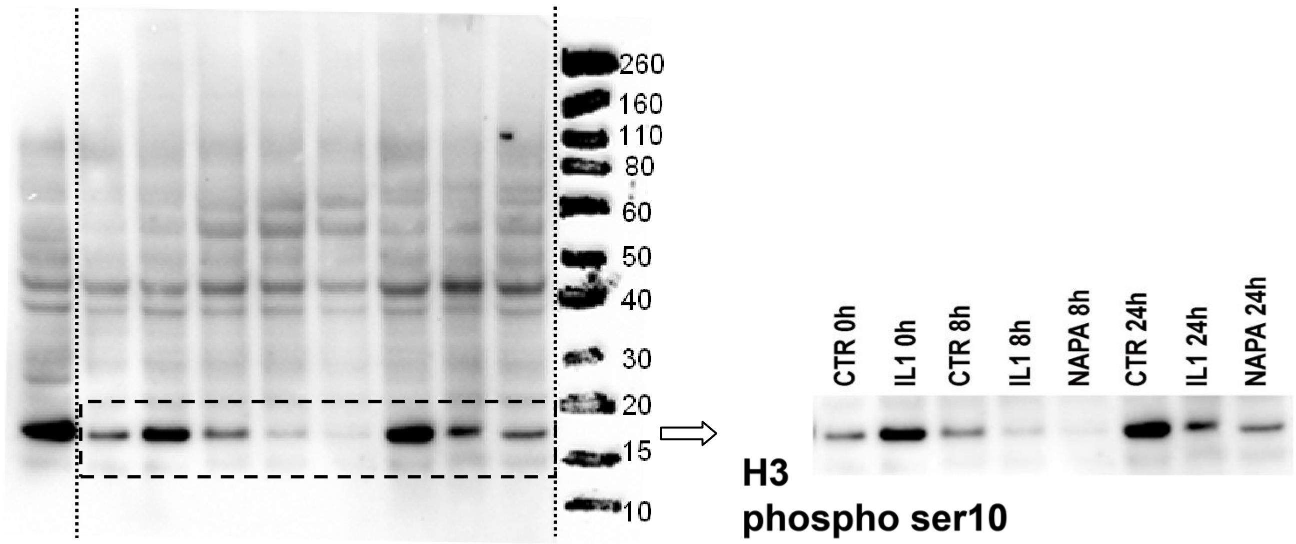
260
160
110
80
60
50
40
30
20
15
10
CTR 0h
IL1 0h
CTR 8h
IL1 8h
NAPA 8h
CTR 24h
IL1 24h
NAPA 24h
H3
phospho ser10

D

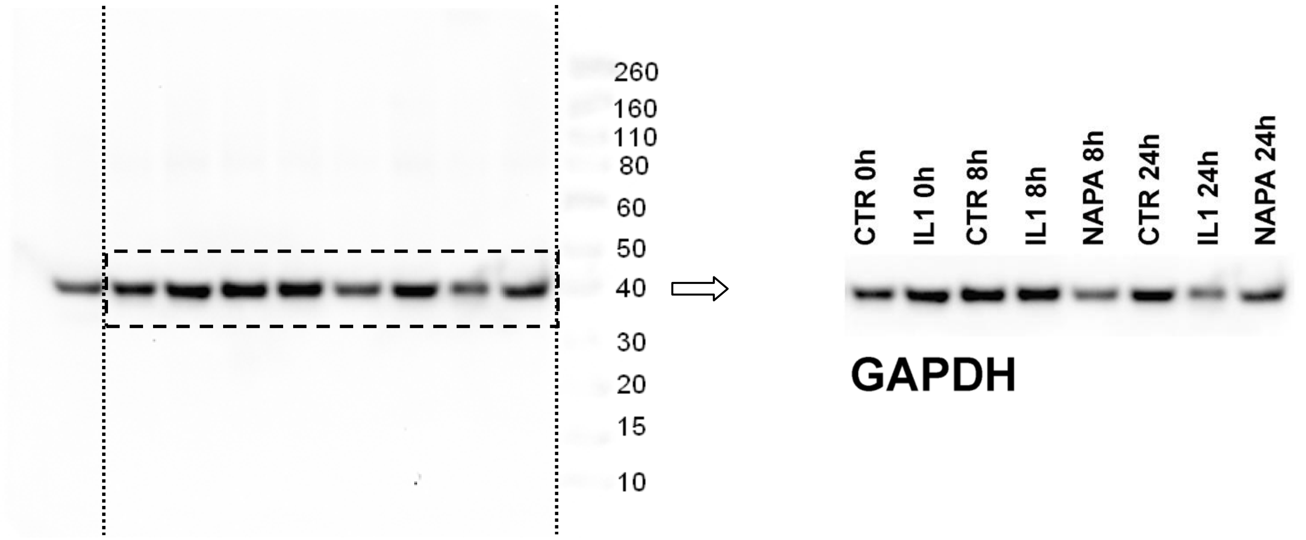
260
160
110
80
60
50
40
30
20
15
10
CTR 0h
IL1 0h
CTR 8h
IL1 8h
NAPA 8h
CTR 24h
IL1 24h
NAPA 24h
GAPDH

Supplementary Figure 6: Full blots used to derive the ADAMTS5, p105-p50, H3 phosphorylated serine 10 results shown in Figure 2, 4 and 5 of the main manuscript.

To obtain these results, cultures of chondrocytes treated exactly as those used for gene expression evaluation were lysed as described in Materials and Methods of the main manuscript, using volumes of the lysis buffer adjusted in order to load two gels with the lysate volumes equivalent to 160000 chondrocytes per lane for each of the 8 conditions: CTR time 0h, IL-1β time 0h, CTR time 8h, IL-1β time 8h, IL-1β+NAPA time 8, CTR time 24h, IL-1β time 24h, IL-1β+NAPA time 24h.

In these gels samples were run with NuPAGE MOPS along with Novex Sharp Pre-Stained Protein Standards. After protein transfer, the lanes containing the standards were cut from the membrane containing the samples. To assess the molecular weight of western blot stained bands the pre-stained bands of the marker were highlighted by mean of a Glow Writer pen (http://divbio.com/glow-writerpen.aspx) and at the end of western blotting, the lanes containing the Protein Standards were juxtaposed to the original membrane and images were taken using the CCD camera acquisition system of a ChemiDoc Imaging Systems apparatus (BioRad).

More in detail, to avoid interference with previous results, one membrane was dedicated for the western blot of the phosphorylated serine 10 of histone H3(17kDA), p105-50 (105kDa and 50 kDa) and GAPDH (37 kDa), and the other membrane was dedicated for the detection of ADAMTS5 (75kDa). Since the lysate volumes were exactly the same the GAPDH signal we showed was that obtained with the membrane used for most antigens.

The dashed rectangles indicates the bands included in the crops, and the crops used to assemble Figure 2, 4 and 5 are reported on the right. In the full blots, dotted lines separate the portions of the membranes used to obtain the results presented in Figure 2, 4 and 5.

A: Full blots used to derive the original ADAMTS5 results shown in Figure 2 of the main manuscript. The dashed rectangle indicates the bands included in figure 2.

B: Full blots used to derive the original p105-50 results shown in Figure 4 of the main manuscript. The dashed rectangle indicates the bands included in figure 4.

C: Full blots used to derive the original H3 phosphorylated serine 10 results shown in Figure 5 of the main manuscript. The dashed rectangle indicates the bands included in figure 5.

D: Full blots used to derive the original GAPDH results shown in Figure 2, 4 and 5 of the main manuscript. The dashed rectangle indicates the bands included in figure 2,4 and 5.
